# The Combination of CA125 and NSE Is Useful for Predicting Liver Metastasis of Lung Cancer

**DOI:** 10.1155/2020/8850873

**Published:** 2020-12-09

**Authors:** Chu-Feng Wang, Sheng-Jia Peng, Rong-Qiang Liu, Ya-Jie Yu, Qian-Min Ge, Rong-Bin Liang, Qiu-Yu Li, Biao Li, Yi Shao

**Affiliations:** ^1^Department of Ophthalmology, The First Affiliated Hospital of Nanchang University, Jiangxi Province Clinical Ophthalmology Institute, Nanchang, 330006 Jiangxi, China; ^2^Department of Hepatobiliary Surgery, The First Affiliated Hospital of Guangzhou Medical University, Guangzhou, 510220 Guangdong, China

## Abstract

**Purpose:**

Liver metastasis is the final stage of cancer progression and is associated with poor prognosis. Although numerous indicators have been identified as having prognostic value for lung cancer and liver metastasis, liver metastases are still not diagnosed by imaging in many patients. To provide a more accurate method for clinical prediction of liver metastasis, we analyzed multiple factors to identify potential predictive factors for liver metastasis of lung cancer.

**Methods:**

Patients first diagnosed with lung cancer between 2002 and 2016 (*n* = 1746) were divided into two groups, with and without liver metastasis. Serum concentrations of calcium, carcinoembryonic antigen (CEA), cancer antigen-125 (CA125), cancer antigen-153 (CA153), carbohydrate antigen-199 (CA199), cytokeratin fraction 21-1 (CYFRA21-1), total prostate-specific antigen (TPSA), and neuron-specific enolase (NSE) were analyzed in both patient groups.

**Results:**

There was no significant difference in age or sex between the two groups. CA125 and NSE were significantly associated with liver metastasis. Compared with CA125, NSE was more specific, while it was less sensitive (*P* < 0.001). Further analysis of NSE concentrations was conducted in patients with non-small-cell lung cancer and indicated that NSE concentration differed significantly between those with and without liver metastasis (*P* = 0.023). We conducted analysis with NSE and CA125 combined, resulting in acceptable sensitivity (51.2%), specificity (72.6%), and area under the curve (0.64) values; sensitivity and area under the curve values were higher than those for individual factors, while specificity was higher than that for CA125.

**Conclusions:**

The combination of CA125 and NSE can assist prediction of liver metastasis of lung cancer, providing improved diagnostic accuracy.

## 1. Introduction

Lung cancer is one of the most common types of cancer worldwide. The disease can develop multiple, complex metastases that are difficult to access a favorable prognosis [1, 2]. There are two major histological classifications of lung cancer: non-small-cell lung cancer (NSCLC) and small-cell lung cancer (SCLC). NSCLC accounts for 80% of all lung cancers, comprising squamous carcinoma, adenocarcinoma, and large-cell carcinoma, and is associated with 5-year survival rates less than 15% [3, 4]. SCLC is characterized by rapid development and growth and early metastases [5], comprising almost 15% of total lung cancers [6]. Treatment for SCLC is often limited, particularly when the metastasis spreads to the liver [7].

Many lung cancers are associated with one or more distal metastases, which are responsible for 90% of patient mortality [8]. The liver, which benefits from an abundant blood supply, is prone to be affected via the blood circulation [9]. Tumor staging and personalized therapy are generally achieved by imaging examination that contributes to TNM staging (Union for International Cancer Control, 7^th^ version) [10]. Although various promising medical imaging methods, such as computed tomography (CT) and magnetic resonance imaging (MRI), have greatly and effectively improved metastasis detection rates, they still have some limitations. For example, occult micrometastases and some infiltrating liver lesions cannot be detected by imaging methods, and only morphological changes, such as nodules or density alteration, lead to suspicion of a metastatic mass. Further, some liver metastases which rapidly progress to fatal acute renal failure in patients with SCLC can only be detected by postmortem autopsy [5]. In addition, once metastasis is detected, the metabolism and breakdown of cytotoxic drugs may make local treatment of the liver difficult [9]. Even after effective surgery, the 5-year survival rate for patients with early-stage NSCLC is merely around 70%, which is inadequate [11]. Further, the results of surgery and chemotherapy are unsatisfactory for most patients with advanced lung cancer. Together, all these factors contribute to the low five-year survival rate for NSCLC. Of NSCLC tumors, 20%–40% eventually progress to liver metastasis [12], which is considered a negative prognostic indicator for patients with NSCLC [13, 14]. Hence, to increase survival rates, it is necessary to improve prediction of the risk and the presence of liver metastasis. Many researchers have sought independent indicators with prognostic value for lung cancer staging, including micrometastasis [15], detection of tumor gene expression [16], and serological markers. However, the results have been controversial and inconclusive [17–21]. Moreover, serological indicators with high specificity and sensitivity for liver metastasis of lung cancer have rarely been reported. Predicting liver metastasis of lung cancer, prior to the presence of an imaged mass, would be of significant benefit for determining prognosis and formulating personalized therapy.

In the present study, we analyzed these 8 serum factors after treatment, including calcium concentration, carcinoembryonic antigen (CEA), cancer antigen-125 (CA125), cancer antigen-153 (CA153), carbohydrate antigen-199 (CA199), cytokeratin fraction 21-1 (CYFRA21-1), total prostate-specific antigen (TPSA), and neuron-specific enolase (NSE), to clarify their prognostic value in predicting liver metastasis in 1746 patients with lung cancer. To achieve optimal prediction accuracy, we also assessed functions of combined NSE and CA125 that were significantly associated with liver metastasis, which presented higher specificity and sensitivity.

## 2. Patients and Methods

### 2.1. Patients

Patients included were all diagnosed with primary lung cancer between 2002 and 2016 and had at least one site of metastasis. Patients had no treatment, including surgery or chemotherapy, before being admitted to the hospital. Patients with primary liver cancer were excluded. Primary lung cancer was diagnosed by pathological examination of specimens obtained by surgical resection or biopsy. CT and MRI were used to diagnose secondary metastasis. This research was supported by the Medical Research Ethics Committee of the First Affiliated Hospital of Nanchang University. All subjects signed informed consent and agreed to take part in this study.

### 2.2. Study Design

Clinical data, including age, sex, time of diagnosis, histopathological subgroup of primary lung cancer, site of metastasis, and treatment, were collected from the medical records of study participants. We also investigated eight tumor markers after their treatment, as follows: calcium, CEA, CA125, CA153, CA199, CYFRA21-1, NSE, and TPSA.

### 2.3. Statistical Analyses

We used an independent *t*-test to evaluate the significance of differences in age, sex, and tumor marker expression levels between the liver metastasis (LM) and nonliver metastasis (NLM) groups. An independent *t*-test was also used to evaluate differences in tumor markers between patients with hepatic metastasis (HM) and nonhepatic metastasis (NHM). Binary logistic regression models were then constructed to determine independent risk factors for LM. Receiver operating characteristic (ROC) curves were also plotted, and area under the curve (AUC) values were calculated. Excel 2010 software was used to calculate cutoff values, sensitivity, and specificity of risk factors. Values of *P* < 0.05 indicated statistical significance. All data were analyzed using SPSS 22.0 (SPSS, IBM, USA) and Excel 2010 (Excel, Microsoft, USA) software.

## 3. Results

### 3.1. Demographic and Clinical Characteristics

**Figure 1:**
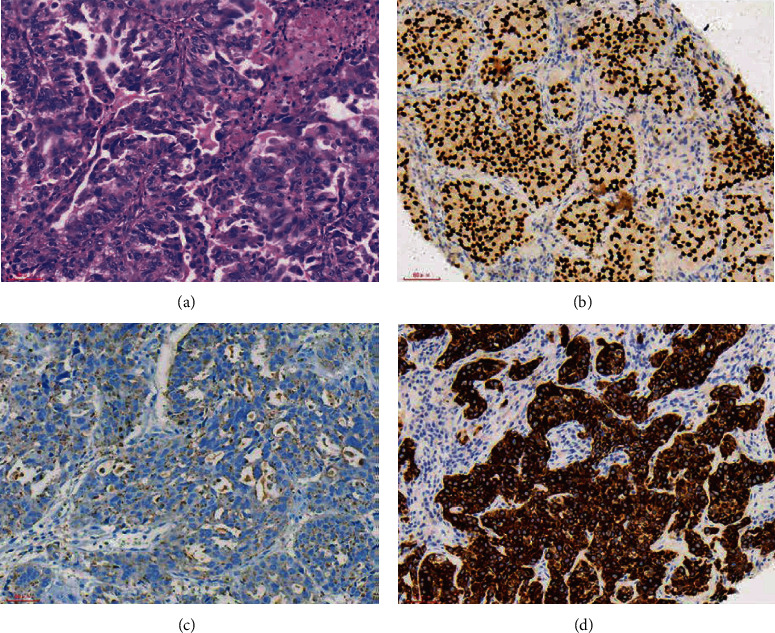
The HE staining and IHC images from lung cancer patients with liver metastasis. (a) Lung cancer (HE ×200). (b) TTF-1(+) (SP ×200). (c) NapsinA(+) (SP ×200). (d) CK7(+) (SP ×200).

**Figure 2:**
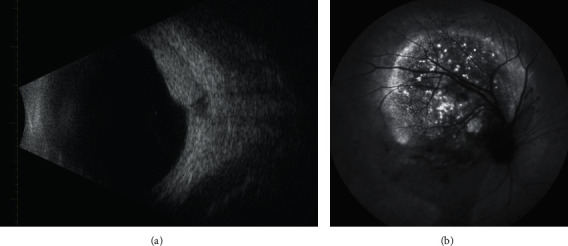
Examples of patients with LM seen on FFA and eye B ultrasonic. Abbreviations: LM: liver metastasis; FFA: fluorescence fundus angiography.

**Figure 3:**
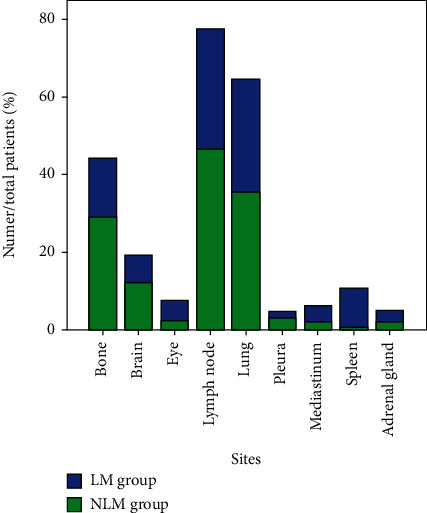
Other metastatic sites in the LM group and NLM group. Notes: the blue bar indicates the LM group while the green bar represents the NLM group. Abbreviations: LM: liver metastasis; NLM: nonliver metastasis.

The total number of participants was 1746, 172 of whom had liver metastasis (LM group), while the remainder did not (NLM group). Patient clinical and pathological parameters are presented in Table 1. The majority of included patients were male (77.33%), and the average ages were 61.3 ± 12.3 and 59.9 ± 11.0 years in the LM and NLM groups, respectively. There was no significant difference in age or sex between the LM and NLM groups (*P* > 0.05, *t*-test). The most common type of lung cancer was adenocarcinoma (40.70% and 48.98% in the LM and NLM groups, respectively), followed by squamous cell carcinoma (33.14% and 32.27%) and SCLC (16.28% and 11.31%). Metastatic sites other than the liver in the LM group were the lymph node (30.81%), lung (29.07%), and bone (15.12%), with corresponding values in the NLM group of 46.44%, 35.32%, and 28.97%, respectively. More details are provided in Table 2 and Figures 1–3.

### 3.2. Risk Factors for Liver Metastasis in Patients with Lung Cancer

To determine whether the eight selected tumor markers can be used to discriminate liver metastasis from other types of metastasis, we compared their levels in the LM and NLM groups. The results indicated that calcium, CA153, CA199, CYFRA21-1, and TPSA levels did not differ significantly between the two groups (*P* > 0.05); however, CEA, CA125, and NSE levels were all markedly increased in the LM group compared with the NLM group (*P* < 0.05). Analysis using a binary logistic regression model indicated that NSE and CA125 could be considered independent factors for prediction of liver metastasis in patients with metastatic lung cancer (*P* < 0.001) (Tables 3 and 4).

### 3.3. Assessment of the Predictive Value of CA125 and NSE Using Cutoff, AUC, Sensitivity, and Specificity Values

**Figure 4:**
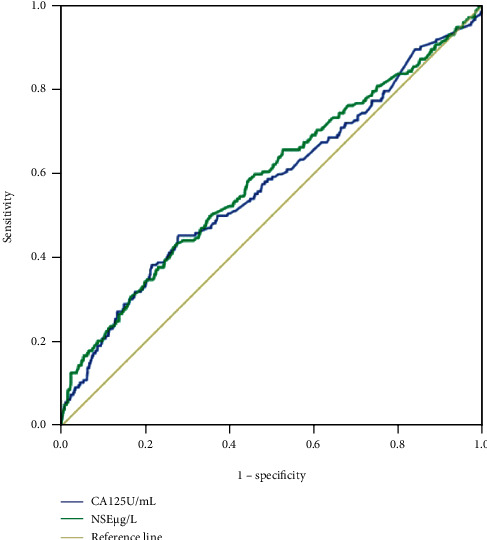
The ROC curves of different factors to predict LM in primary lung cancer. Notes: CA125 and NSE were performed as a single factor in detecting LM in ROC curves. Abbreviations: CA: carbohydrate antigen; NSE: neuron-specific enolase; LM: liver metastasis; ROC: receiver operating characteristic.

**Figure 5:**
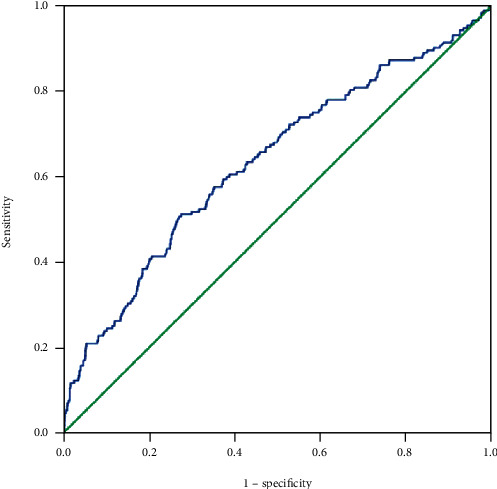
The corresponding cutoff value divided patients into two parts in the LM group and NLM group, respectively. Notes: in the LM group, 45% of patients' CA125 levels were above the cutoff value (53 U/mL) while only 28% of participants were higher than 53 in the NLM group. 42% of patients' NSE levels were above the cutoff value (23.4 U/mL) while only 27% of that in the NLM group. Abbreviations: CA: carbohydrate antigen; NSE: neuron-specific enolase; LM: liver metastasis; NLM: nonliver metastasis.

**Figure 6:**
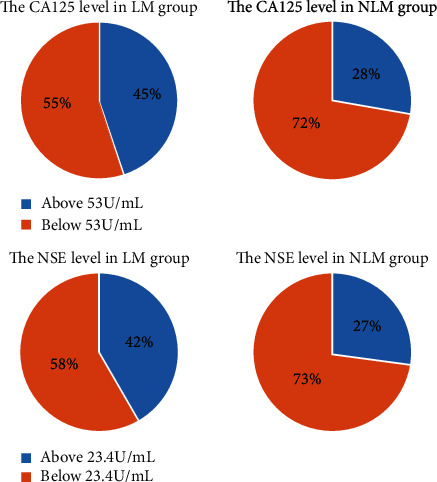
The ROC curve of the combination of CA125 and NSE for diagnosing LM in primary lung cancer. Abbreviations: CA: carbohydrate antigen; NSE: neuron-specific enolase; LM: liver metastasis; ROC: receiver operating characteristic.

To analyze the predictive value of CA125 and NSE, ROC curves were plotted (Figure 4). The cutoff values for CA125 and NSE were 53.0 U/mL and 23.4 *μ*g/L, respectively, while the AUC values were 0.57 and 0.59 (Figure 5). The corresponding sensitivity and specificity values for CA125 were 45.3% and 72.1%, respectively, while those for NSE were 43% and 72.9% (Table 5). Additionally, the ROC curve and AUC values were determined for the two factors combined and the resulting AUC value was higher than those for either individual marker (Figure 6). More details are presented in Table 4. These results indicate that the combination of CA125 and NSE may be useful for prediction of liver metastasis in patients with metastatic lung cancer.

### 3.4. Comparison of NSE Levels in Patients with Intrapulmonary Metastasis without SCLC

NSE levels are generally elevated in patients with SCLC, and participants with intrapulmonary metastasis included some with SCLC; therefore, it was necessary to eliminate the influence of SCLC to determine the predictive value of NSE for the remaining patients.

**Figure 7:**
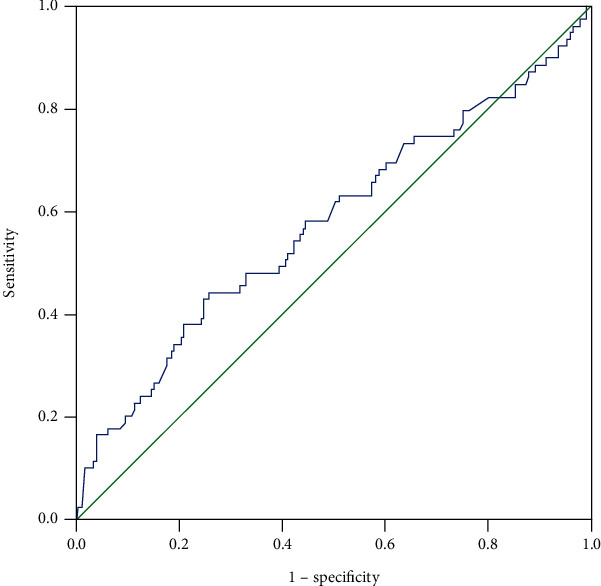
The ROC curve of the combination of CA125 and NSE for diagnosing LM in primary lung cancer. Notes: NSE was performed as a single factor in detecting HM in the ROC curve. Abbreviations: NSE: neuron-specific enolase; HM: hepatic metastasis; ROC: receiver operating characteristic.

The total number of participants included in this part of the analysis was 927, after exclusion of patients with SCLC and liver metastasis. Of these, 848 (604 males and 204 females) had no liver metastasis while 66 males and 13 females had metastasis to the liver. NSE levels differed signsuificantly between the HM and NHM groups (independent *t*-test, *P* = 0.032). Further, AUC values indicated that NSE had predictive value for patients with NSCLC metastasized to the liver (Tables 6 and 7 and Figure 7). Therefore, diagnosis of primary lung cancer with liver metastasis can be more reliably predicted using combined CA125 and NSE serum levels. Hence, the combination of CA125 and NSE may be an indicator that can predict the presence of liver metastasis in patients with lung cancer.

## 4. Discussion

In 2007, there were 1.5 million prevalent cases of lung cancer worldwide, representing 12% of new tumors, and the number of deaths from lung cancer accounted for 17.6% of cancer mortality, making it the most important cause of cancer death [22]. Previous studies have reported no difference in rates of liver metastasis among different types of lung cancer, consistent with our findings. Our data indicated that of all histological types, adenocarcinoma accounted for the majority of cases of liver metastasis, reflecting that NSCLC accounted for the largest proportion of lung cancer cases. None of the other histological types showed a significant difference in rates of metastasis.

Although patients with early lung cancer have a 70% survival rate after appropriate surgical treatment, many may already have developed distal metastasis, which cannot be detected by imaging methods. Tumor-related side effects can also cause patient death. Liver metastasis of lung cancer was detected in 5.8% of surviving patients; however, the proportion with liver metastasis increased significantly following postmortem examination [22]. In our study, the proportion of patients with liver metastasis was approximately 11%, which was higher than previous reports. However, 64.5% of 251 patients with SCLC were reported to have liver metastasis [23]. These findings demonstrated the disadvantage of the low sensitivity of imaging methods. Based on clinical data, patients with tumors of a higher degree of differentiation and TNM stage were more likely to have distant metastasis, and patients with liver metastasis had an average survival period of 4 months [24]. Examination of tumor metastasis determines staging; however, traditional TNM staging appears to be insufficient to facilitate prognosis and to determine treatment. It has been suggested that the following three factors should be added to TNM staging: (1) number of involved sites, (2) number of metastatic foci per involved site, and (3) diameter of each metastatic focus. Prediction of metastasis is helpful for more accurate tumor staging, which could improve treatment efficacy and increase survival rates [25].

The expression of metallomatrix protease (MMP) by the organ microenvironment and tumor cells is crucial for the organ-specific selectivity of lung cancer cells; hence, whether lung cancer exhibits multiple organ heterogeneity may depend on the presence of MMPs [26]. Metastasis involves many sequential steps that all of them must be completed for successful spread to a new site. Since we do not know the destination of new metastasis, more attention should be focused on these sequential cellular processes, to facilitate prediction and prevention of lung cancer metastasis to specific organs.

Identifying patients at high risk of metastasis has always been challenging, and indicators that can predict metastasis accurately and efficiently are urgently required. We aimed to identify safer and more convenient as well as nonorgan invasive and economical methods for patients with metastatic diseases. In a recent study, we analyzed a series of possible factors that may contribute to predicting liver metastasis in patients with primary lung cancer.

Tumor markers are bioactive molecules synthesized by specific types of cancer that can be detected when released into body fluids [27]. Ideal tumor markers act as complementary methods for imaging examination, which can be used to assess the efficacy of chemotherapy, and are regularly used to evaluate prognosis; however, due to low specificity and sensitivity, many markers are not strongly correlated with disease, and rather than being applied for rapid diagnosis, it could only be considered a guide.

NSE is a 78 kDa gamma homodimer glycolytic enzyme that is widely expressed in endocrine neurons and especially neuroendocrine tissues. Further, NSE is overexpressed in neuroblastoma and SCLC [28], which are often derived from differentiated neural crest tissues. Although NSE is a relatively common tumor marker used for evaluation of SCLC in the clinic, it may also be useful for other applications [29]. In general, NSE levels correlate with SCLC staging before treatment. Pinson et al. reported that serum NSE levels were higher in SCLC patients with poor prognosis than in those with better prognosis [30]. Further, pretreatment serum NSE levels were associated with brain metastasis of advanced NSCLC [31]. Together, the above evidence indicated that NSE was likely related to the degree of tumor malignancy and could be closely associated with specific tumor metastasis. Tumors with high levels of NSE were primarily derived from neural crest cells (NCCs), which underwent epithelial-mesenchymal transition, involving extremely high activity of the beta-catenin signaling pathway to facilitate cell metastasis and invasion. Inhibition of the beta-catenin pathway reduced levels of gastric cancer metastasis [32]. In contrast to the findings of the present study, van de Pol et al. reported that changes in NSE concentration were associated with tumor recurrence and with indiscriminate metastasis, rather than metastasizing to a specific site [33]. In our study, NSE levels strikingly differed between patients with metastatic lung cancer with or without liver metastasis, which was of great significance for patients with occult metastasis.

CA125 is a cleavage peptide of MUC16 [34], which is a member of the mucin family of glycoproteins that is often used as a tumor marker [35]. Initially, CA125 was identified as a biomarker for gynecological tumors [36], and elevated concentrations were also detected in patients with cervical adenocarcinoma [37]. In recent decades, a role for CA125 in lymph node and peritoneal metastases was reported [38]. CA125 was found in mesothelial cells of the peritoneum, pleura, and epithelium of the fallopian tubes and endometrium [39], and serum levels of CA125 can be raised in response to inflammation or metastasis in these sites [40]. Moreover, CA125 was higher in patients with liver or peritoneal metastasis of pancreatic and gastric cancer [41], and patients with higher baseline concentrations were more likely to present recurrence during the postoperative period [42]. Currently, it was difficult to detect small tumor metastases by imaging; however, CA125 was considered an important serological indicator. CA125 levels were closely related to worse prognosis and metastasis development, possibly attributable to promoting tumor cell proliferation and inhibiting antitumor immune responses [43, 44]. Although levels of CA125 were associated with tumor metastasis, the organ specificity of this marker was controversial among researchers. Concentrations of CA125 have been reported not to be associated with prediction of tumor metastasis to specific organs, such as the bone or liver [45]. In our study, CA125 expression levels differed markedly between the LM and NLM groups, with acceptable sensitivity (45.3%) and a specificity of 72.1%. The cutoff and mean CA125 values were also important data, and serum concentration was informative. Hematogenous tumor dissemination was more likely in patients with higher CA125 concentrations, and CA125 was an independent indicator at the time of analysis, with a cutoff value of 13.65 U/mL [46]. In our study, the CA125 cutoff value was much higher (53 U/mL), supporting the hypothesis that liver metastases were largely hematologic. Various studies demonstrated that CA125 levels were significantly related to liver metastasis in other cancers [47, 48], and our analysis supported the value of serum CA125 for prediction of liver metastasis of lung cancer. Moreover, we discovered that the specificity and AUC values for NSE were higher than those for CA125, although neither was completely satisfactory. We did not calculate the influence of sex on CA125. It was thought that increases of CA125 in male patients were less clear, relative to those in females, likely due to the influence of female-specific organs, with the ovaries in particular having a large effect on serum CA125 concentration [49]. Hence, the results of our study were suboptimal, and the statistical analyses conducted were not sufficiently comprehensive; nevertheless, our data had value for prediction of liver metastasis, since minor fluctuations in serum levels of markers may indicate the likelihood of metastasis.

Data from the present study provided convincing evidence that the combination of NSE and CA125 can precisely predict liver metastasis. First, we found that NSE levels were significantly different between patients with all histological types of lung cancer with liver metastasis and without liver metastasis (*P* < 0.001), even after excluding the specific effects of SCLC on NSE levels. Patients with high serum NSE levels were more likely to have liver metastasis, suggesting that NSE concentration may be a useful supplementary examination during early diagnosis. Although NSE levels differed significantly between the two groups, NSE alone was insufficient for predicting the presence of liver metastasis because its specificity was lower than 90%. Second, serum CA125 concentrations were much higher in lung cancer patients with liver metastasis than in those without, suggesting that CA125 was also associated with lung cancer liver metastasis. After demonstrating that these two indicators were significantly associated with liver metastasis, we focused on their sensitivity for liver metastasis, to exclude the influence of other metastatic locations on the data. Third, we found that both factors had relatively high specificity for liver metastasis, particularly NSE. Notably, the specificity and sensitivity of the two markers combined were higher than either individual factor, indicating that more accurate prediction can be achieved by this combination. The higher prediction accuracy of the combination was related to the specificity and area under the curve (AUC) values, while sensitivity remained relatively unsatisfactory. Previous reports did not evaluate the role of this combination in liver metastasis of lung cancer; hence, our data represented an important contribution.

Our study had several limitations. We did not investigate or document patient survival, preventing comparison of survival rates for patients with metastases at various sites. In addition, given the long time span during which the data were collected, errors both by human operators and of laboratory inspection probably occurred, compromising the accuracy of the data; however, significant differences were detected. Further, we did not assess the effects of sex, age, or other factors on serological indicators. Moreover, we were unable to determine the sequence of the multiple lung cancer metastases; hence, further focus on the cause and effects of metastasis was required for facilitating a more detailed investigation.

In conclusion, the combination of NSE and CA125 can more accurately predict liver metastasis of lung cancer than either factor separately.

## Figures and Tables

**Table 1:** The clinical characteristics of patients with primary lung cancer.

Characteristics	LM group (*n* = 172)	NLM group (*n* = 1574)	*P* value
Gender^a^			
Male	133 (72.2%)	1137 (77.3%)	0.155
Female	39 (27.8%)	437 (22.7%)	
Mean age^b^	61.3 ± 12.3	59.9 ± 11.0	0.105
Histopathological type			
Squamous cell carcinoma	57 (33.1%)	508 (32.3%)	
Adenocarcinoma	70 (40.7%)	771 (49.0%)	
Large-cell carcinoma	3 (1.7%)	22 (1.4%)	
Small-cell lung cancer (SCLC)	28 (16.3%)	178 (11.3%)	
Poorly differentiated	11 (6.4%)	57 (3.6%)	
Other NSCLC	1 (0.6%)	25 (1.6%)	
Unknown	2 (1.2%)	13 (0.8%)	
Treatment			
Surgery	17 (10.0%)	269 (17.1%)	
Chemotherapy	111 (64.5%)	1000 (63.5%)	
Radiotherapy	17 (9.9%)	185 (11.8%)	
Symptomatic treatment	42 (24.4%)	355 (22.6%)	
Others	13 (7.6%)	85 (5.4%)	

Abbreviations: LM: liver metastasis; NLM: nonliver metastasis; NSCLC: non-small-cell lung cancer. ^a^Chi-squared test was performed. ^b^Independent *t*-test was performed. *P* < 0.05 revealed a significant difference.

**Table 2:** The other sites of metastasis in primary lung cancer.

Sites	LM group	NLM group
Bone	26 (15.1%)	456 (29.0%)
Brain	12 (7.0%)	190 (12.1%)
Eye	9 (5.2%)	36 (2.3%)
Lymph node	53 (30.8%)	731 (46.4%)
Lung	50 (29.1%)	556 (35.3%)
Pleura	3 (1.7%)	46 (2.9%)
Mediastinum	7 (4.1%)	31 (2.0%)
Spleen	18 (10.5%)	4 (0.25%)
Adrenal gland	5 (2.9%)	32 (2.0%)

Abbreviations: LM: liver metastasis; NLM: nonliver metastasis.

**Table 3:** Comparison of tumor markers between the LM group and the NLM group.

Tumor marker	Calcium (mmol/L)	CEA (ng/mL)	CA125 (U/mL)	CA153 (U/mL)	CA199 (U/mL)	CYFRA21-1 (ng/mL)	NSE (*μ*g/L)	TPSA (ng/L)
LM group	2.23 ± 0.23	104.05 ± 233.30	164.30 ± 360.52	32.14 ± 70.44	150.75 ± 731.14	15.96 ± 40.17	48.43 ± 77.88	1.75 ± 1.80
NLM group	2.24 ± 0.26	58.46 ± 307.30	79.20 ± 183.50	22.30 ± 33.24	55.21 ± 528.00	11.69 ± 35.79	26.54 ± 38.83	1.81 ± 4.18
*P* value	0.771	0.02	0.003	0.072	0.097	0.142	<0.001	0.867

Note: independent *t*-test was performed. *P* < 0.05 revealed statistical significance. Abbreviations: CEA: carcinoembryonic antigen; CA125: cancer antigen-125; CA153: cancer antigen-153; CA199: carbohydrate antigen-199; CYFRA21-1: cytokeratin fraction 21-1; NSE: neuron-specific enolase; TPSA: total prostate-specific antigen; LM: liver metastasis; NLM: nonliver metastasis.

**Table 4:** The binary logistic regression model between the LM group and the NLM group.

Tumor marker	CEA (ng/mL)	CA125 (U/mL)	NSE (*μ*g/L)
*B*	0.000	0.001	0.006
Exp(*B*)	1.000	1.001	1.006
*P* value	0.104	<0.001	<0.001

Note: the binary logistic analysis was performed. *P* < 0.05 means a significant difference. Abbreviations: *B*: coefficient of regression; CEA: carcinoembryonic antigen; CA: carbohydrate antigen; NSE: neuron-specific enolase; LM: liver metastasis; NLM: nonliver metastasis.

**Table 5:** The cutoff value, sensitivity, specificity, and AUC of CA125, NSE, and CA125+NSE in detecting the LM in metastatic lung cancer.

Factor	Cutoff value	Sensitivity	Specificity	AUC	*P* value
CA125	53.0 U/mL	45.3%	72.1%	0.57	0.001
NSE	23.4 *μ*g/L	43%	72.9%	0.59	<0.001
CA125+NSE		51.2%	72.6%	0.64	<0.001

Note: the sensitivity and specificity were calculated at the point of Youden's index. *P* < 0.05 indicates statistical significance. Abbreviations: AUC: area under the curve; CA: carbohydrate antigen; NSE: neuron-specific enolase; LM: liver metastasis.

**Table 6:** The expression level of NSE between the HM group and the NHM group.

Factor	HM group	NHM group	*P* value
NSE	40.4 ± 63.6	24.6 ± 34.0	0.032

Note: independent *t*-test was performed. *P* < 0.05 revealed statistical significance. Abbreviations: NSE: neuron-specific enolase; HM: hepatic metastasis; NHM: nonhepatic metastasis.

**Table 7:** The cutoff value, sensitivity, specificity, and AUC of NSE in predicting the HM in metastatic lung cancer.

Factor	Cutoff value	Sensitivity	Specificity	AUC	*P* value
NSE	23.2	43.3%	73.9%	0.58	0.023

Note: the sensitivity and specificity were calculated at the point of Youden's index. *P* < 0.05 indicates statistical significance. Abbreviations: AUC: area under the curve; NSE: neuron-specific enolase; HM: hepatic metastasis.

## Data Availability

All data are in the manuscript and can be obtained from the corresponding author.

## Abbreviations

LM: Liver metastasis
NLM: Nonliver metastasis
NSCLC: Non-small-cell lung cancer
CEA: Carcinoembryonic antigen
CA125: Cancer antigen-125
CA153: Cancer antigen-153
CA199: Carbohydrate antigen-199
CYFRA21-1: Cytokeratin fraction 21-1
NSE: Neuron-specific enolase
TPSA: Total prostate-specific antigen
AUC: Area under the curve
HM: Hepatic metastasis.

## Supplementary Materials

Supplementary Materials The markers' levels of each patient.
