# Supplementary material for: The Combination of CA125 and NSE Is Useful for Predicting Liver Metastasis of Lung Cancer
Source: Dis Markers. 2020 Dec 9;2020:8850873. doi: 10.1155/2020/8850873 (PMC7746448; doi:10.1155/2020/8850873)
Supplement: Supplementary Materials — The markers' levels of each patient. [file 8850873.f1.pdf]

Calcium (mmol/L)		CEA (ng/mL)		CA-125 (U/mL)		CA-153
LM	NLM	LM	NLM	LM	NLM	LM
2.32	2.24	5.1	9.2	411.3	91	181
2.21	2.31	4	81	6	66	21
2.23	2	38.39	3	942.2	8	32
2.81	2.31	277	1.27	253	13.48	8.35
2.17	2.12	79.12	2	222.1	9	14
2.01	2.2	9.94	1	9	97	11
2.18	2.36	40.65	1.08	206.2	9.6	54.22
2.17	2.31	8.36	4	236.8	8	11
2.07	2.13	4	4	9	6	9
2.44	2.05	12	13.78	13.22	22	7.67
2.27	2.28	20.08	55	272.85	8	23
2.39	2.33	81.87	17.04	87.38	49.58	11
2.17	2.26	63.62	24.99	11.87	451.4	8.5
2.43	2.2	2.67	53	2.81	21	20.91
2	2.25	172	1.9	9	905.4	2
2.02	2.32	218	228.4	9	58.46	11
2.11	2.3	0.36	1	107.7	21	4.63
2.32	2.42	574	12	12	9	21
2.48	1.96	16.33	9.1	9	56.29	9
2.26	2.1	1.89	1	22	21	21
2.1	2.2	54	1	224	54	15.6
2.18	2.12	209.4	7	2227	60	450.2
2.23	2.02	32	66.37	89	57	3
2.21	1.96	217.2	8.35	26.06	91	9
2.16	1.83	481.9	2.91	770.2	21.2	14
2.1	2.3	859	23	32	14	24.2
2.28	2	5.74	609	10.23	14	7.93
2.2	2.27	10.39	23	58.31	22	9
2.43	2.37	4	1.21	21	15.17	9
2.16	2.17	0.93	1.17	18.79	8.26	8
2.2	2.55	105	23	1200	6	13
2.16	2.12	12.59	3.82	144.92	30.15	23
2.41	2.08	13.69	2.02	1129	15.15	2
1.89	2.32	79	68.11	1200	48.09	500
2	2.15	64.29	21	124.4	9	9
2.5	2.19	32	46.71	8	31	9
2.1	2.4	1	15	97	33	9
2.07	2.4	1	12	174	7	8
1.98	2.4	2.89	24	22	9	4
2.08	2.17	1.74	34	10.25	22	13.74
2.59	2.42	18.72	1	121.4	66	38
2.14	2.32	10.37	6.21	26.87	44.91	9
2.01	2.23	55	284.6	76	29.59	11
1.68	2.33	172.46	38.63	197.31	26.55	74
2.11	2.28	1050	1	7	120	3
2.26	2.21	20.41	16.01	22.93	14.45	12
2.2	2.47	6	44.31	28	29.5	9
2	2.27	105	0.47	82	6.67	13
2.1	2.14	32	1.69	13	12	11

2.28	2.19	123.5	1.43	1854	34.42	8
2.01	2.32	1	11.36	9	23	158
2.02	1.28	216.8	43	1221	9	40.71
2.32	2.48	209	1	6	91.97	32
2.16	2.24	54	44.04	71	15.88	21
2.01	2.15	720	1.56	1200	25.62	21
2.34	2.22	29	3.95	122	33.73	47
2.1	2.35	2	1.51	41	15	7.54
2.42	2.26	32.42	1.44	21	42.53	9
2.41	2.18	12	28	9	166	2
2	2.5	511	1	9	18	32
2.02	2.3	2.32	44	5.34	143	4.69
2.22	2.34	1.68	4	530.1	18	21
2.4	1.92	27.63	32	12	6	32
2.08	2.08	245.5	23	72.15	77	9
2.24	2.43	46.45	51.57	168.9	62.18	21
2.3	2.66	28.63	23.56	9	19.27	9.28
2.35	2.09	1.88	31.96	121.6	172	4
2.02	2.3	77.66	405	31.6	271	9
2.52	2.24	2.23	1.19	225.3	528.4	9
2.43	2.58	1571	45	7	7	9
1.35	2.47	4.27	4	10.59	91	9
2.38	2.49	16.59	252.5	56.8	6	56.8
3.5	2.32	1	3.61	22	1193	121.8
2.26	2.3	3	1	54	57	21
1.9	2.02	3	6.72	91	13.32	11
2.5	2.13	0.56	2.13	272.8	36.6	121
2.09	1.98	7.43	7.47	21	23.43	13
2.43	2.07	428.3	25.37	488.4	52.49	201.9
2.43	2.45	568.2	2.66	467	59.73	231
2.43	2.34	49.15	67.59	111.2	22	8
2.26	2.14	3.69	2.58	45.53	102	17.46
2.15	2.2	40.19	5.2	11.94	9	21
2.43	2.4	181	12.3	38	44	14
2.14	2.1	3.67	45	125.4	64	8
2.5	2.2	43	41	21	32	2
2.3	2.7	40.9	1050	30.73	221	9
2.26	2.31	21.68	11	29.49	8	22
1.93	2.32	66	160.2	21	7	9
2.08	2.2	4	42	257	21	59.65
2.25	2.16	24.56	0.05	14	22.45	21
2.1	2.04	51	1050	76	317.2	2
2.41	2.1	32	1.42	22	9	11
2.18	2.17	1221	106.4	372.4	218.3	12
2.22	2.42	1.11	3	24.72	7	21
2.15	2.34	0.81	8.85	1.62	11.51	25.9
2.58	2.13	32	425.8	9	1213	8
2.05	2.51	465.7	1.86	60.85	99.5	9
2.75	2.11	17.24	48.41	660.35	3.05	500
2.42	2.12	1.16	2.97	128.3	19.95	8
2.19	2.24	54.76	5.83	43	32	21

2.06	2.16	253.9	113.9	59.62	5	50.84
2.32	1.8	822.3	0.41	39.33	37.07	16.02
2.13	2.15	4.32	4.56	12	22	4.32
2.02	2.4	21	3	9	54	4
2.3	2.3	2.07	3	170.9	693	9
2.3	2.19	1050	41	143	9	12.66
2.3	2.15	38.26	0.54	69.78	35.92	19
2.06	2.53	695.8	2.3	28.31	54	8
2.18	2.38	2.22	105	96.44	327.58	13
2.5	1.96	2.15	14.91	41.05	67.94	32
2.19	2.56	34	11.46	9	52.63	17.89
2.87	2.3	1	89	9	182	1.17
2.56	2.22	1.67	3.57	16.68	27.7	5
2.48	2.2	0.7	0.49	3	12	21
2.35	2.1	105	2	3	168	21
2.17	2.47	5.57	55.27	15.95	124.8	62.45
2.37	2.47	62.64	12	4	73	7
2.15	1.91	2.48	21	4.38	24	12
2.14	2.06	24.68	14	10.03	42	21
2.38	2.25	0.57	2	8.02	7	21
2.17	2.07	1.01	2	1200	30	45.37
2.17	2.8	6.65	3.87	7	9	1.93
2	2.8	32	2.37	33	907.5	11
2.09	2.36	131.5	45.13	149	192.8	12
2.23	2.01	105	3.79	9	9	21
2.3	2.07	2.4	2.66	73.05	47.47	11
2.46	2.58	0.02	4.44	62	14.22	0.17
3.1	2.11	1	3.12	23	46	28.71
2.16	2.29	4.77	21	16.22	37	11.66
2.12	2.19	3.79	2.49	53.25	5.26	12.72
2.51	2.37	31	23	34	7	3
2.46	2.13	28.45	32	15.27	43	14
2.32	2.13	7.68	1	30.38	9	8.04
2.19	2.86	4	16	74	54	8.54
2.28	2.3	164.8	47.53	612	29.2	36
2.2	2.33	31	12	54	9.27	12
2.67	2.55	80.94	37.13	28	7.36	11
2.51	2.31	12	54	32	91	21
2.1	2.48	5	91	108	61	32
2.2	2.49	4.57	0.92	77.6	92	96.78
2.29	2	1.83	5.1	7.5	302.2	12.3
2.16	2.04	21	0.65	24	17	11
2.33	2.14	2.46	105	15.14	76	12
2.3	2.2	13	4	66	41	13.23
2.3	2.3	45	5	189	21	156
2.26	2.2	21	1	9	231	11
2.16	2.14	299	43	156	43	13
2.1	2.26	1	9	244	6	28
2.17	2.23	4.9	4	188.4	33	91
2.19	2.32	4.67	149.8	21	1104	11
2.34	2.15	30.99	1.83	5	9.45	22.7

2.23	2.1	105	1	16	71	2
2.11	2.25	432	4	53.16	51	25.99
2.3	2.21	4.52	14.58	13.18	18.37	9
2.1	2.38	10.5	18.88	53	14.58	3.2
1.87	1.96	1.51	2.85	6.84	16.11	8
2.28	2.15	12	2.26	8	63.85	12
2.23	2.23	71.15	1.43	2289	204.2	9.8
2.02	2.25	6.1	5.38	71.01	4	13.29
2.14	2.15	15	12.3	44	51	31
2.08	2.3	50	1	19	170	16
1.91	2.3	105	53	21	8	11
2.24	2.7	46	2	9	9	7
1.89	2.1	42	5	321	43	9
1.87	2.28	6.47	1050	37.92	224	12.1
2.1	1.75	52	3.24	54	247.9	46.09
2.5	2.13	12	3	32	58	13
2.26	2.29	38.13	21	123.6	64	182.9
2.3	1.98	5	2	32	887	12
2.22	2.13	25	19	9	425	43
2.01	2.47	12	37	76	44	11.87
1.92	2.6	23	7.97	261	59.89	9
	2.59		3.01		198.7	
	2.37		2.25		86	
	2.25		55.08		402.1	
	2.29		1.85		151.7	
	2.55		0.99		75.77	
	2.38		1.6		30.86	
	2.49		4.39		20.95	
	2.42		73.71		6	
	2.47		50		4	
	2.27		4		8	
	2.39		2		25	
	2.32		1		6	
	2.36		29.79		86.86	
	1.92		34		6	
	2.48		61.18		47.21	
	2.42		1.97		11.63	
	2.01		2.64		103.2	
	2		23.21		66	
	2.18		1.7		84.73	
	2.3		4		11	
	1.98		5.2		13	
	2.1		21		14	
	2.3		12		21	
	2.2		33		6	
	2.2		42		33	
	1.73		63		330	
	2.23		5		10	
	2.15		4.65		82.87	
	2.21		4.76		61.81	
	2.14		1		43	

2.69	13.22	17.37
2.67	10.91	16.86
2.41	151.7	7
2.57	7.44	29.28
2.51	580	191.8
2.47	0.97	10.79
2.4	6.5	29.33
2.58	1.7	6.59
2.09	3	9
2.26	26.73	59.49
2.44	1.23	8
2	4	8
2	23	6
2.43	3	62
2.39	3.78	675.56
3.03	4.02	26.09
2.14	726.12	18.8
2.14	4.59	148.7
2.59	1.12	42.66
2.09	70.46	49.3
2.2	62	542.2
1.88	2.04	68.78
2.27	43.21	32
2.12	745	8.63
2.25	4.29	39.14
2.23	9.77	49.84
2.4	2	6
2.55	53	21
2.25	65	32
2.13	19	240
1.94	32	13
2.23	8.17	29.62
2.14	1	135
2.37	31	7
2.32	1.64	7.55
2.35	97.26	24.86
2.32	1.01	20.16
2.34	12	32
2.26	4.45	6.16
2.19	14	6
2.06	51	7
2.31	1	21
2.28	12	6
2.35	70.36	10.54
2.46	22	96
2.23	4	37
2.7	1	96
2.26	2.1	32
2.43	2.01	24
2.08	2.3	87
2	3.34	41

2.13	1.04	22
2.28	39.02	257.1
2.1	3	1200
2.3	1	85
2.2	12	29
2.3	42	9
2.2	1	4
2.85	4	8
2.77	4	33
2.61	4	13
2.26	17	7
2.13	6	6
2.25	2	5
2.31	4	65
2.43	10.1	52.97
2.47	6.72	78.99
2.62	5847	12
2.45	4.24	96.5
2.12	56.93	396.61
2.19	1.1	3
1.97	4.9	31
2.55	4	76
2.31	12	32
2	3.3	112
2.6	12	22
2.46	2.55	16.01
2.2	2.51	17.23
2.34	5.39	12.43
5.13	64.89	24.32
2.38	7.76	8.59
2.31	1.63	4
1.96	1.91	22
2.41	4.22	8.12
2.44	96.1	22.53
2.5	32	8
2.4	138	118
2.2	4	23
2.3	2	49
2.3	32	21
2.56	2.18	4.42
2.3	23	65
2.3	3	7
1.63	1	127
2.28	17	3
2.13	32	7
2.15	32	21
2.2	2	488
2.46	5.11	8
2.27	9	121
2.2	5	14
2.31	477.3	15.86

2.29	1.42	23.6
2.46	1.79	115.7
2	2.1	7
2.41	18.97	65.43
2.18	4	5
1.67	3.39	45.12
2.78	10.77	29.08
2.16	71.4	112.3
2.34	1.16	9.37
1.96	6.28	19.19
2.13	1.84	19
2.39	1.82	11.54
2.24	0.57	23.21
2.37	2.12	168.5
2.09	157.6	52
2.4	4.35	21.47
2.14	2.98	21
2.34	2.8	24.66
2.19	6.29	50.08
2.5	1	18
2.3	34	23
2	32	21
2.4	12	21
2.6	1	43
2.3	1050	21
2.3	12	21
2.4	3	43
2.18	18.68	162.2
2.4	21	32
2.3	14	9
2.2	12	43
2.15	23	9
2.1	11	8
2.13	0.99	44.47
2.07	2.45	24.68
2.22	15.95	12.76
2.51	3	3
2.23	11	48
2.17	12.9	11.76
2.41	1.82	4
2.39	37.28	390.3
2.44	5.3	86.66
2.19	4.57	9
2.26	10.21	226.16
2.14	1.33	21
2.33	1.28	2.5
2.09	1	116
2.3	18	59
2.07	57.45	22
2.13	1.85	8
2.25	4.11	15.15

1.89	2.14	44.48
2.01	3.62	9.88
2.26	189.4	41
2.24	2.45	4.69
2.18	1.95	45.9
2.27	3.66	21.97
2.33	6.72	12
2.31	3.92	7
2.15	3.02	9.24
2.54	2.27	57.99
2.11	3	31
2.03	7.19	19.92
2.3	17	6
2.15	4.53	67
2.2	1	7
2.2	35	26
2.3	3	32
2.2	6	9
2.7	2	32
2.2	9	6
2.3	98	8
2.1	2	122
2.12	2	9
2.35	11.52	37.81
2.2	330.2	49.27
2.21	21	4
2.48	211.5	127
2.31	245.79	487.07
2.06	1	36
2.58	3.33	22
2.01	7.77	741.4
2.27	1	9
2.15	4	7
2.35	13.82	13.12
2.2	5	15
2.12	1.09	18.4
2.31	2.96	38.78
2.37	10.73	22.11
1.98	23.8	62.63
2.05	21.57	19
2	1.49	16.64
2.12	2.26	25
2.18	0.95	10.25
2.09	1.41	8.7
2.4	4.43	13
2.31	54.32	7
2.17	16.64	73.84
2.15	0.97	6.94
2.21	21	12
2.2	3.76	12
2.1	4	37

2.3	25	54
2.3	4	24
2.2	42	12
2.07	12	9
2.34	11	9
2.15	27	9
2.36	5.84	21.11
1.85	12	9
2.21	4	43
2.21	1	168
2.6	24	21
2.08	1.8	15.27
2.55	3.07	178.9
2.24	3.36	23.96
2.08	1.48	11
2.02	1	8
2.37	8.52	13.39
2.4	2.27	6
1.88	1.85	343.87
2.53	83.81	398.1
2.32	7.41	16.19
2.14	2.14	201.2
1.96	0.71	189.9
2.1	2.26	8.13
2.06	137.3	1506
1.64	4.83	10.42
1.97	1.92	29.27
1.71	8.88	22
2.04	1.91	5.68
2.09	14.02	32
2.23	4.38	10.61
2.35	9.85	11.45
2.11	29.88	12
2.16	12.36	21
2.11	4.57	17.3
2.05	4	16
2.04	32.51	61
2.06	0.67	31
2.3	32	22
2.4	1	21
2.3	25	7
2.5	14	44
2.5	21	7
2	1	30
2.49	101	197
2.25	1	9
2.11	1	13
1.62	2	11
2.13	43	22
2.45	8	66
2.16	57	590

2.2	288.7	225.2
2.35	12	7
2.33	2	32
2.15	4.37	9.77
2.51	19.3	43.11
2.18	171.6	6
2.42	1.08	5.56
2.24	2.53	33.98
2.11	30.07	8
2.5	30.49	17.71
2.54	2.2	15.84
2.61	19.27	15.3
2.4	77.28	66.8
2.23	12	8
2.28	4	6
2.01	0.68	72.64
2.7	2.51	9
2.15	5.17	348.9
2	3.6	10.82
2.72	4.86	4.41
2.25	30.21	62
2.62	42	108
2.2	21	8
2.21	3.62	19.21
2.36	2.36	21
2.03	312	22
1.93	12.5	17.69
1.83	66.67	386.4
2.14	0.84	11.24
2.15	2.96	17.26
2.55	2.65	5.73
2.3	25	6
2.3	43	34
2.4	32	21
2.3	2	54
1.92	1	32
1.79	4	11
2.24	21	49
2.19	4.15	42.36
2.35	201.3	10.15
2.56	4	32
2.21	4	9
2.07	1	77
2.37	1.7	8
2.43	85.94	22.55
2.21	42.98	7
2.21	48.12	16.42
2.38	3.29	8
2.59	4.27	34.23
2.55	7	8
2.46	23	61

2.14	6.88	488.83
2.3	3424	77
2.37	8.02	85.44
2.47	1.12	45.29
2	25.78	12
2.13	2.68	23.35
2.21	2.53	14
2	4.28	20.82
2.17	6.15	32
2.28	80.8	198.1
2.52	144	207
2.4	4.9	10.71
1.76	5.43	21
2.25	1.92	7.79
2.02	3.72	153
2.13	6.8	19.99
1.95	1.29	1.69
2.18	11.33	32
2.17	2.66	12
2.4	25	8
2.2	1	4
2.3	14	7
2.3	4	9
2.2	1	36
2.22	3	66
1.93	4	11
2.2	8	179
2.54	18	21
2.32	52	76
2.14	3	43
1.99	4	33
2.2	33.66	65.18
2.04	13.73	144.2
2.01	24	7
1.99	3	156
2.2	510	124.6
2.4	1.8	7
2.36	4.87	357.9
2.4	30.17	44.2
2.48	35.37	31.1
2.32	2.53	7.05
2.08	1.24	20.76
2.31	1.3	8
2.09	4	8
2.38	17.3	4
2.12	54	9
2.4	4.59	320.09
2.45	1	199
2.11	1000	127.2
2.03	3.49	42.14
1.67	1.73	86.63

2.16	3.97	26.56
2.14	20.57	22.03
2.15	19.43	35.85
2.5	13.25	26.77
2.24	5566	280.8
2.3	3.29	22.92
2.37	2.66	21
2.14	2.99	1970
2.41	1.93	20.38
2.1	3.35	17.11
1.9	2	23
2.14	7	33
1.82	8.91	7
2.27	12	22
2.15	23	23
2.21	2.17	42.87
2.24	14	7
2.51	1	25
2.47	12	76
2.42	1.34	8.54
2.2	3.66	21.05
2.44	6.66	48.65
2.11	3.4	36.07
2.35	6.11	30.7
2.34	4	9
2.38	1.78	31.17
2.43	3.78	15.89
2.38	11.96	31.95
2.26	4	9
2.58	1.24	750
2.23	1	7
2.33	4.35	74.28
2.16	1.3	16.8
2.28	4	64
2.1	1.18	31.37
2.41	3.01	18.38
2.01	6.65	13
2.18	0.8	149.9
2.15	8.06	35
2	4.54	21
2.12	2.01	26.32
2.33	8.56	10.11
2.48	2.87	69.62
2.4	32	21
2.2	3	14
1.92	3.23	6
2.3	9	43
2.3	12	7
2.3	23	21
2.3	1	56
2.3	1050	1200

2	14	228
2.2	552	349
2.08	69	9
1.78	4	22
2.16	106	22
2.83	22	6
2.07	4.36	81.58
2.29	2	21
2.23	3	14
2.2	1	9
2.1	9.52	15.11
2.19	6.6	12
2.35	3.03	23.66
2.34	2.43	10.62
2.13	3.19	11.73
2.53	413.9	22.73
2.1	4	8
2.28	3.09	7.11
2.24	4	9
2.16	4.02	12.81
2.39	13.9	36.41
2.5	1.53	13.33
2.8	19.69	1200
2.04	43.95	123
1.87	8.68	887.7
2.4	67.41	77.15
2.12	2.57	17.32
2.51	2.24	6.77
1.89	2.9	32
2.23	3.32	12
1.94	2.63	24.13
2.34	10.95	32.57
1.93	3.17	19.97
1.95	4.09	104.1
2.05	13.38	85.02
2.24	6.13	29.43
2.16	3.42	22
2.25	63.23	184.3
2.2	23	12
2.3	1	32
2.3	41	9
2.6	12	22
2.3	12	8
2.1	105	586
2.32	6	71
2.33	2	7
2.31	45	54
2.28	14	4
2.25	4	12
2.12	4	51
2.3	78.34	42.64

2.24	65	21
2.28	230.3	9
2.47	43.89	30.23
2.29	13.08	21.01
2.56	4	9
2.34	2.12	33.84
2.2	5.32	12.14
2.01	23.6	29.88
2.02	1	46
2.47	1	40
2	2.17	19.71
2.42	2.31	9
2.43	3	7
2.15	0.96	87.03
2.12	169.5	739
1.95	135	106.3
2.24	77.55	15
2.23	43.97	21.19
2.43	4.02	54.14
2.33	37.83	196.3
1.95	26.37	32
1.78	150.8	544.2
2.37	36.13	44.34
2.09	1.37	12.09
2.12	23.1	21
2.36	1.98	25.54
2	330.4	439.5
2.43	4	23
2.13	49.46	85.91
2.3	1	16
2.3	2	112
2.2	3	9
2.4	1	8
2.3	31	22
2.33	43	34
2.3	25	43
2.17	73.97	8
2.16	15	23
2.2	17.95	9
2.21	88	143
2.2	5	53
2.63	89	205
2.29	205	174
2.7	1	224
2.57	7.53	36.31
2.46	3.22	35.78
2.06	521.1	113.5
2.1	150.9	31.6
2.39	3.48	31.93
2.2	1.15	122.3
2.41	1.26	24.49

2.54	3.64	47.03
2.26	34	9
2.34	34	6
2.14	4	7
2.36	2.34	8
2.25	1050	725
2.1	34	23
2.39	2.81	12.6
2.01	2.25	11.2
2.19	1.68	17.65
2.09	2.26	68.53
2.1	2.5	6.56
2.17	3.17	12
1.91	13.74	12.23
2.07	2.41	12
1.91	2.94	31
2.2	1	21
2.2	21	32
2.3	32	3
2.4	2	9
2.4	31	34
1.92	23	77
1.88	2	7
2.61	119	9
1.91	4	17
2.02	1	64
2.62	189.3	135.9
2.45	1.63	155.1
2.25	7.36	11.88
2.35	406.2	56.79
2.38	1.79	21.36
1.86	12.21	7
2.63	2.12	87
2.41	2.75	5.05
2.44	1.73	4.74
2.45	87.91	10.86
1.95	13.55	294.3
2.05	45.44	53
2.27	20.77	322.6
2.29	157	122.1
2.52	106.2	345.9
2.16	6.91	8.14
2.22	3.24	14.53
1.94	0.98	50.25
1.88	0.74	24.07
2.23	20.41	6
2.14	1.49	58.09
2.3	34	21
2.3	2	36
2.8	23	9
2.18	4.95	40.06

2.24	3.16	5.07
2.34	12	6
1.82	3	27
2	14	8
2.27	2.72	13.64
2.32	3.64	6
2.52	17.06	28.48
2.43	3.13	29.83
2.35	1.23	40.27
2.29	25.27	99.78
2.29	8.67	8
2.17	1.62	123.33
2.15	4	32
2.36	3.62	87
2.3	5.59	36.95
1.87	12	32
2.51	36.66	438
2.37	2.69	30.65
2.45	1.6	22.56
2.14	2.51	21
1.9	43.31	49.81
2.03	3	68.49
2.17	3.61	23.8
2.16	0.73	8.91
2.38	52.02	21
2.42	5.06	90.16
2.2	3.25	115.2
2.15	260.9	48.38
2.16	5.34	25
2	12	17
2.1	21	21
2.5	43	6
2.4	2	9
2.2	42	23
2.8	12	54
2.5	1	47
2.46	42	32
2.25	3	43
2.29	106.8	25.08
2.24	0.92	38.67
2.16	7.95	15.47
2.1	66	9
2.24	2.23	51.47
2.26	1.61	32.42
2.37	1.93	23.31
2.4	3.03	13.79
2.45	0.79	14.62
2.58	8.36	14.59
2.26	1.43	192.3
2.32	1.69	12.69
1.93	1	8

2.01	4	91
2.22	31.2	9.26
1.77	8	7
2.14	2.87	15.86
2.02	73.15	181.73
2.14	12.43	66.59
2.17	3.49	49.95
2.31	13	21
2.52	3.27	10.64
2.03	191.9	22.9
1.89	2.12	24
2	7.7	94.96
2.07	5.59	21
2.08	6.7	10.22
1.95	1.37	141.4
2.03	34	22
2.15	4	52
2.6	105	7
2.3	3	32
2.5	2	4
2.3	32	32
1.88	1	66
2.24	5	8
2.21	35	9
2.27	1	80
2.5	69.9	126.1
2.57	3	43
2.23	1	56
2.55	21	21
2.14	28	9
2.12	11	9
2.11	3	98
2.17	13	8
2.44	3.26	14.78
2.47	1.01	182.25
2.85	4.29	22.85
2.02	2.3	33.62
2.31	12	24
2.66	0.64	231.05
2.19	2.67	31.88
2.16	2.14	31.77
2.3	45.64	189
2.03	3.45	12.97
2.31	3.23	3.13
2.33	2.43	12.63
2.12	6.54	42
2.06	57.49	45.04
2.15	7.23	13.57
2.26	14.64	10.44
2.15	5.34	21
2.03	2.46	24.14

2.03	2.62	36.04
2.15	5.12	250.6
2.25	4.32	43
2.1	6	35
2.2	21	32
2.8	12	8
2.2	53	24
2	18	124
2.28	42	43
1.91	4	9
2.41	468.5	56.09
1.88	31.87	300.2
2.34	1	6
2.37	31	9
2.29	105	29
3.29	38.54	45.52
2.44	221	7.29
2.81	3.95	59.51
2.34	14.88	66.62
2.14	5.47	61.47
2.03	16	5
2.41	2.44	119.1
2.28	2.87	84.53
2.42	9	68.27
2.4	2.34	7.16
2.24	1.75	181.57
2.05	8	7
2.01	31	32
2.14	51	45
2.22	2.82	7.47
2.32	0.52	8.35
1.9	72	68
2.4	12.75	11.04
2.18	23.37	11.98
1.92	9.67	36.61
2	276.8	113.6
1.59	1138	18
2.42	8.99	92.52
1.74	2.39	7
2.38	1.94	12
2.47	7.31	35.83
3.4	14	22
2.3	2	250
2.3	8	49
2.3	69	15
2.2	9	431
2.02	5	1054
2.25	86	535
2.7	13	43
2.34	135.8	37.87
1.97	16.41	65.77

2.27	6.4	155.3
2.07	12	9
2.53	3	9
2.23	5	9
2.29	3.27	9
2.24	7.6	14.66
2.26	21	7
2.26	8.75	26.16
2.34	4.06	6.46
2.2	3.66	21
2	22	6
2.13	35.12	184.5
2.24	5.98	32
2.15	6.91	70
2.06	3.95	23.71
2.26	34.24	12
2.2	44	7
1.99	9	13
2.1	42	31
2.18	873.2	139.7
2.26	8.04	33.82
2.33	4	44
2.62	4.19	51.09
2.39	3.88	10.22
2.31	2.2	64.78
2.3	2.46	22
2.98	3.84	14.64
2.32	3.21	9
2.33	24.02	716.6
2.21	38	1200
2.03	4.73	8.04
2.29	268.9	21.55
2.23	6.21	130.1
2.22	4.32	39.73
2.08	2.68	47.24
2.25	1.99	5
2.3	24	9
2.3	21	4
3.01	1	24
2.13	4	7
2.04	1	13
2.16	4.63	136.5
2.21	4	9
1.87	1	16
2.3	4.67	19.38
2.52	5.03	27.3
2.35	6.87	7
1.9	3.35	6
2.35	2	19
2.05	25	6
2.13	2.43	8

2.71	2.79	142.34
2.3	1	99
2.02	2.05	63.2
2.02	12.93	249.1
2.13	7.6	91
2.06	51.02	518.7
2.22	13.19	30.11
2.17	7.12	13.62
1.99	2.7	22
1.96	24.05	42.27
2.3	1050	1200
2.2	2	15
2.1	14	9
1.96	2	9
2.2	4	416
2.42	126.2	51.49
1.89	11.76	120.1
2.38	5	12
2.25	31	22
2.24	12	23
2.26	28.35	150.9
2.22	13.68	28.28
0.49	6.69	5.92
2.18	1	13
2.77	31	32.97
2.33	31	21
2.72	2.08	8
2.11	216.17	48.38
2.22	1.2	31.81
2	12	8
2.11	8.53	54
2.17	36.34	63.03
2.26	3.84	8.33
2.15	19.93	15
2.09	186.8	22
2.41	6.24	37.25
1.71	7.2	15
2	1.93	21
2.11	3.81	22.91
2.15	5.28	11.35
2.09	8.64	3.21
2.13	2.45	42
2.3	2	53
2.3	105	32
2.3	2	52
1.91	1	10
1.9	23	32
2.42	19	35
2.34	14	18
2.33	7.15	31.5
2.15	7	174

2.13	2	34
2.25	2	126
2.42	1.03	23
2.16	3.18	135.9
2.37	55	21
2.02	2.06	74.46
2.11	2.07	4.77
2.01	1.17	18.2
2.29	3.7	13
2.3	4.67	4
2.12	15.2	32
2.5	2	15
2.3	1	49
2.2	17	21
2.4	2	9
2.7	3	32
2.3	3	34
2.29	6.18	35.69
2.19	107.3	16.46
2.41	1	41
2.41	2.44	32
2.26	4.07	15.78
1.98	15	1022
2.12	1.99	7.8
2.41	26	40
2.79	2	3
2.32	104.5	31.95
2.38	23.82	8.97
2.22	3.44	62.92
2.38	272.5	26
2.05	3.24	61.09
2.14	7.1	7.25
2.13	1.67	144
2.15	9.99	7.91
2.03	18.85	100.6
1.9	7	46
2.1	2	32
1.9	3.13	13.06
2.3	26	3
2.26	8	52
1.77	7	10
2.29	125	8
2.1	235.5	21
2.1	2.54	52.53
2.08	32	32
2.01	21	21
2.35	2.14	8.86
2.22	43.61	24.1
2.37	1.57	32.49
2.14	3.58	6
2.31	4	3

2.14	8.41	88.95
2.01	2.17	24
2.13	3	43
2.65	2.28	92
3.91	12.35	19.85
2.2	11	314.32
2.32	176.9	627.5
1.89	1.22	85.98
2.37	3.07	17.53
2.24	4.55	27
2.13	25.71	27.64
2.5	21	9
2	16	31
2.18	14	7
2.25	11.14	7
2.3	82.69	86.32
2.16	25.56	19.47
2.4	2.98	11
2.55	1.41	11.28
2.05	11.14	65
2.32	19.59	25.13
2.32	37.74	318.1
2.12	5.67	15.41
2.08	2.26	30.79
2.05	97.07	62.45
2.31	2.75	22.25
2.3	624.5	548.3
2.3	9	9
2.2	28	9
1.83	12	21
1.77	31	32
2.52	48.33	39.82
2.07	210.6	75.92
1.85	2.1	75.06
1.93	4.98	35.37
2.48	809.7	28.91
2.35	7.93	11.03
2.01	5.9	7
2.01	373.4	188.3
2.4	2.54	41
2.06	54	23
2.3	23	9
1.94	12	6
2.09	21	7
1.84	12.64	53.53
2.66	50	4
1.92	2.6	53.83
2.48	105	9
1.9	8	6
2.28	3.76	31.7
2.26	2	28

2.28	78.94	50.23
2.2	1.2	41.16
1.8	3.71	42.32
2.48	0.92	8
2.16	2.1	8.38
2.38	1.69	12
2.15	13.69	293.3
2.39	17.29	19.17
2.2	40.6	29
2.4	1.62	14.57
2.4	5.18	925.06
2.08	1.86	17.52
2.32	2.12	8
2.24	1	159.48
2	10.52	67.53
1.95	3	22
2.1	53	21
2.2	1	8
2.3	31	8
2.04	41	11
1.9	21	51
2.08	1	9
2.06	23	13
2.1	32	15
2.38	1.18	30.55
2.28	1	54
2.3	3.76	38
2.02	12.53	250.5
2.3	11	11
2.29	2.46	27.05
2.01	10.35	22.8
2.32	1.34	21
1.94	0.99	97.06
1.98	283	186
2.38	12	24
2.37	16	21
2.29	18.96	23.54
1.56	6.62	434.3
2.17	4	21
2.35	7	8
2.35	14.19	9
2.3	64	65
2.37	18.74	40.78
2.14	1.17	16.47
2.08	0.69	75.53
2.3	47	160
2.3	10	121
2.1	1000	27
2.16	1	22
2.23	1	71
2.36	17.68	30.36

2.38	23	4
2	1.25	1200
2.2	31	28
2.03	5.99	429.8
2.3	54	9
2.24	4	14
2.27	69.43	6.94
2.13	0.71	328.4
2.13	36.07	9
2.9	1	9
2.3	25	46
2.2	40	112
2.3	154	27
2.34	2	109
2.38	12.3	12
2.57	27.86	841.2
2.45	3	44
2.4	1	22
2.1	1	15
2.3	8.68	26.42
2.16	58.8	185.6
2.18	11.25	68.78
2.2	6601	2656
2.3	23	41
2.1	81	9
2.3	12	6
1.93	2.4	40.01
1.82	1.47	82.02
2.39	70.62	43
2.2	60	6
2.1	160	415
2.32	31	9
2.29	37.01	98.79
2.24	1.48	22.29
2.16	1.77	21.28
1.98	43	9
2.1	21	7
1.45	39	129
2.29	50.88	81.54
2.47	1.63	10.96
2.06	105	18.64
2.46	31.23	28
2.46	11.1	7
2.22	45.57	16.37
2.21	21.95	39.72
2.2	3.51	18.25
2.22	1	93.97
2.22	26.86	35
2.33	1.66	21
2.28	1.12	10.42
2.1	1	1200

2.1	53	26
2.3	6	9
2.3	42	8
2.1	4	9
2.21	65	77
2.38	47	334
2.49	12	9
1.98	4	21
2.41	92.31	323.3
2.07	4.95	21
2.16	86.86	294.6
2.29	3.07	32.76
2.22	43.39	18.29
2.28	73.4	25.96
2.25	9	100
2.17	14	21
2.1	25	3
2.1	12.54	86.34
2.36	50.72	93.7
2.41	243.5	25.72
2.19	13	5
2.18	59.67	10.76
2.13	3.23	19.97
2.4	54.97	223.8
1.92	14	7
2.25	1	33
2.1	283.21	462.68
2.2	1.91	116.86
2.48	135.7	26.15
2.14	417.29	317.15
2.17	1.07	15.87
1.78	12.5	14.25
2.24	6.9	9
2.21	3	7
2.3	14	24
2.1	65	9
1.91	1	168
2.32	2.37	23.37
2.45	2.51	55.05
2.31	13	37
2.47	4	22
2.48	20.67	21
1.9	917.7	422.1
2.3	5.68	19
2.47	9.62	24.35
2.15	2.15	202
2.13	0.91	86.63
2	2.54	42
2.1	12.3	41
1.9	239	138
2.29	69.69	19.17

2.14	1.8	32
2.2	299	33
1.9	23	44
2.42	31	32
2.25	1.99	274.7
2.2	4.37	48.5
2.22	3.6	77
2.17	3	6
2.45	4.02	13.98
2.39	5.24	7.8
2.29	4	8
1.95	1.4	9.43
2.41	55.83	7.15
2.44	10.42	22.42
2.22	925.99	16.65
2.15	72.1	37.04
2.18	5.21	15
2	4.61	62
2.15	3.54	149.6
2.34	7.7	20.63
2.12	9.13	47.7
2.3	105	9
2.3	3	44
2.3	21	33
1.9	3	6
2.13	31	76
2	1.62	15.8
2.14	32	51
2.18	31	32
2.42	0.93	9.92
2.04	3.02	102.9
2.14	0.26	12
1.96	2.34	123.5
2.38	280.8	109.2
2.05	23.16	15.42
2.28	40.33	32.44
2.24	3.42	38
2.6	34	21
2.19	8.05	8.77
2.4	12	21
2.11	4	64
2.53	8	168
2.12	21	9
2.3	3.24	25.35
2.3	3	8
2.1	1.42	42.85
2.03	3.53	21
2.34	27.26	416.6
2.11	5.46	21
2.4	32	87
2.41	3	43

1.94	21	9
2.53	43	44
2.5	4	610
2.2	8	37.09
2.34	1.27	10.71
2.38	5.61	9
2.42	70.71	22.96
1.91	2.78	26.54
2.32	6.43	77.78
2.06	303.9	169.2
2.16	1	61
2.37	11	634
2.24	3	13
2.13	21	52
2.22	0.66	63.26
2.21	29.33	13.39
2.25	32	9
2.13	8	6
2.43	13	44
2.1	1.49	8.4
2.2	3.13	10.58
2.28	16.15	24.49
2.44	5.58	16.66
2.47	5.68	11.5
2.52	23	7
2.24	4	8
2.07	5.75	46.46
1.89	14.06	116
2.11	27.84	222.7
2.04	28.88	11.38
2.38	12	33
2.38	1000	110.4
2.3	0.71	34.39
2.42	3.23	9.45
2.13	12	7
2.39	3.37	8.97
1.98	22	6
2.23	56.85	63
2.65	2.49	23
2.14	7.25	13.26
2.63	3.22	12.94
2.4	322	755
1.9	14	21
2.2	13	33
2.58	24	75
2	1	6
2.28	0.73	9.7
2.32	76.23	19.76
2.53	24.3	54.81
2.24	4	183
2.16	21	33

2.31	32	23
2.6	9.2	48.03
2.15	2.16	9.06
2.33	228.2	162
2.43	4.39	163.1
2.6	86.06	9
1.92	42	62
2.37	7.72	17.43
2.19	3.02	22
2.21	4.56	14
2.4	2.72	75.56
2.1	2.46	12.1
1.9	321	32
1.9	10	4
2.16	8.06	26.48
2.16	2	186
2.24	4	24
2	12	7
2.29	1219	28.69
2.18	2.02	38.84
2.27	43.61	51.96
2.3	12	32
2.4	4	8
2.53	230	352
2.31	85.03	134.8
2	54	9
2.2	7.59	46.98
1.82	16	7
2.17	7.62	565.08
2.11	461.6	9
2.28	114.6	100
1.96	1.1	37.11
2.25	32	33
2.42	1	69
2.45	2.41	7
2.31	3.24	9
2.2	1050	1200
2.55	3	9
2.14	3.78	21
2.28	3.7	37.83
2.06	2.53	23
2.42	23.4	45
2.23	196	80.93
2.5	597.8	70.49
2.41	4	23
2.41	16.58	17.57
2.16	579.1	68
1.94	1.73	11.42
2.14	7.65	123.5
2.13	295.5	23.45
2.16	2.08	24.25

2.15	22.42	12.66
1.88	4.72	9
2.14	8.67	24.92
2.1	1.62	22.35
2.4	23.4	158
2.3	105	701
2.6	6	21
2.4	12	55
2.47	1.97	7.94
2.39	4	8
2.33	12	7
2.27	1231	1513
2.33	11.64	57.4
2.25	186.2	26.54
2.24	2.8	118.3
2.24	36.48	21
1.99	1.48	23.07
2.25	33.48	52.58
1.44	21	9
2.3	8.02	59.83
2.35	2.31	9
1.79	137.8	115.8
1.87	12.65	155.6
2.16	4.87	32
2.3	29.63	71
2.1	1	10
2.07	43	6
1.95	15	215
2.24	48.2	6
2.39	1396	14.29
2.32	25.24	54
2.03	23	7
2.55	13.72	7
2.13	1.14	33.8
2.2	32	32
1.85	1	28
2.16	43	51
2.02	5.23	44.17
2.31	194	26
2.39	2.4	150.5
2.5	4	23
2.27	8.62	728
2.3	12	31
2.23	53	116
1.97	2	93
2.6	8	6
2.45	212.9	869.2
2.21	24	9
2.3	841.6	21.41
2.15	25.63	88.15
2.15	24.39	45.95

2.09	105	361
2.06	1.47	18.67
1.66	53.75	21.15
2.47	2.39	22
2.2	10	51
2.3	1	8
2.7	12	6
2.3	3	23
2.31	32	43
2.09	4.22	57.99
2.5	1	9
2.36	10.58	22.89
2.32	0.82	36.5
2.19	3.73	224.1
2.27	1.99	14.19
2.3	43.11	198.3
2.1	2.26	13
2.4	26.63	32
2.46	21	13
3	4.32	10.94
2.57	32	8
1.98	37	531
2.1	54	48
2.1	4.2	78.15
2.14	1.44	500
2.46	2.54	52
2.2	34	21
2.4	21	6
2.2	5	487
2.41	11	38
2.1	345.2	13.76
2.16	1.28	48.77
1.77	2.21	18.52
2.09	11	7
2.15	4	9
2.26	105	77
2	13.71	49.91
2.11	53.87	459.3
2.26	2.48	8.36
2.16	5.52	21
2.1	3	21
2.22	402	78
2.35	4	7
2.31	15.55	8.52
2.26	4.73	126.8
2.46	614.55	1169.44
2.2	41	43
2.53	59	51
2.17	32	21
2.49	0.78	52.99
2.36	2.89	32

2.28	7.86	221.66
2.56	3.65	160.56
2.05	260.9	121.5
2.31	3	23
2.12	21	76
2.46	3.8	9.73
2.29	86.44	48.27
2.33	3.23	76
2.06	3	7
1.92	22	9
2.38	2.06	57.8
2.17	17	9
1.69	148.06	554.18
2.23	3.76	9.18
1.84	2.55	87.21
2.3	1	17
2.26	3.4	85.13
2.21	5	105
2.4	105	33.35
2.2	3.04	93.21
2.34	32	32
2	1.69	11.15
2.22	496	117
2.19	9.58	27.15
2.7	23	8
2.32	9.47	45.77
2.13	2.2	45.14
2.39	70.62	15.35
2.07	6	98
2.33	6	105
2.2	8	107
2.12	0.5	6.37
2.01	106	56.57
2.07	6.43	32
2.36	12.39	68
1.82	1	32
2.14	24	43
2.11	1000	252
1.98	22.67	60.76
2.27	39.09	6
2.4	7.28	73.73
2.06	12	55
2.2	12	7
2.04	112.4	5.33
2.51	9.77	6.94
2.03	315.9	304

(U/mL)	CA－199 (U/mL)		CYFRA 21－1(ng/mL)		NSE (
NLM	LM	NLM	LM	NLM	LM
8	9.1	3	2.78	5.02	19.49
21	32	14	2.31	2.23	149.2
7.48	806.1	13	43.91	2.43	69.26
32	61	241.8	4.16	36.06	12.4
9	14	21	21.44	12.25	13.4
6.33	14	6	1.57	1.02	6
9	22.05	31	1.56	1.42	9.43
8	579.5	13	10.15	4.5	25.86
21	32	21	23.12	2.13	16.32
11	13.22	21	1.54	5.82	121.5
9	12	24	2.43	2.27	22.53
162.4	292	18.79	3.44	2.1	18.8
21	6.74	40.06	8.03	5.89	24.72
8.89	21	12	10.22	1.23	9
83.22	12	48.39	24.76	2.56	22.06
361	1835	511.1	1.6	2.72	16.47
8	5.9	3	3.43	5.81	37.07
21	22	15	4.51	23.43	23.63
170.3	714.3	4.54	3.84	5.77	24.55
5	7.08	3	9.89	16.04	370
11	43.52	32	25	2.43	23.4
6	53.94	7	67.4	2.91	17
11	21	4.89	5.81	3.42	14.87
5.28	12.3	5.28	9.58	1.32	165.8
9	246.2	12	1.48	5.62	22.02
12	12	3	3.73	3.44	12
21	6.69	21	1.32	2.01	12.32
139.7	23	21	9.5	5.89	11.33
11	21	4.63	3.21	42.78	23.2
43	3	11	12.23	2.43	21
12	54	3	8.45	1.32	27.19
15.23	180.6	2.17	4.8	9.21	43
33	11	3.76	2.66	7.21	116.1
11	255	4	32.4	2.49	54
12	107.1	11	1.54	3.83	370
21	13	11.74	4.53	6.29	3.21
12	10	21	10.22	5.53	14.87
8	9	32	3.83	23.31	87.39
21	3	8	3.24	2.84	118
21	20.6	22	0.9	8.45	16.77
23	1	9	4.22	12.25	18.8
21	3	62.01	4.2	2.31	13
76.67	574	21.99	16.04	2.32	39.16
4	16.18	14.11	3.83	14.6	11.44
9	117	18	2.01	1.25	20.46
5.65	4.04	8.04	3.41	2.71	8.99
21	3	167.4	1.3	6.03	48.29
14.17	574	7.34	2.43	5.64	10.93
11	8	21	2.1	3.19	22.06

11.87	12	7.11	18.95	2.52	14.96
12.36	32	13	2.84	2.05	32.13
9	175.3	11	256.2	6.27	18.6
77.89	263	7	3.43	1.21	12.18
21.99	44	4.51	3.45	4.53	10.63
7.67	359	6.07	2.49	2.13	17.37
24.29	12	82.96	2.43	20.04	16.76
6	46	32	4.32	34.56	5
6.26	21	21	5.64	5.17	21
2	3	3	4.5	10.7	21.32
11	12	3	8.96	5.24	23.85
14	32	119	4.53	1.4	32
9	72.51	33	32.54	32.1	151.1
8	4	51	7.54	8.45	12.62
21	41.22	21	32.24	234.2	50.07
4.01	21.34	12.22	8.66	4.8	19
236.04	4	13.53	5.64	34.5	12
21	6.83	12	30.31	10.93	18.96
21	11.96	135	1.32	6.54	23.1
8	14.45	3.39	1.32	9.48	13.39
9	11	21	13.57	41.53	13.42
3	7.98	3	1.45	4.22	370
32	12.16	32	11.19	14.55	114
59.81	11	11.29	2.91	47	21
9	3	34	2.32	1.34	370
14.21	1	6.33	4.17	2.89	8.14
0.95	574.2	10.6	2.14	4.65	32.12
11.1	3	22.32	23.1	5.42	23.6
16.16	22	20.54	77.69	3.11	370
21	281.6	15.59	43.2	1.83	34
11	20.21	21	5.45	3.99	20.61
9	35.96	12.6	7.62	4.16	32.33
11	1.56	12	13.22	9.37	15.06
11.66	39	12	6.27	3.24	32.1
8	87.9	11	3.8	1.54	16.95
21	6	32	92.34	1.24	11
45.57	39.3	44	3.11	5.64	37.21
11	10.88	12	2.86	1.4	12.42
21	11	21	2.43	15.54	14
11	601	7	4.29	3.73	14
9.8	12	12	4.2	3.42	9.57
7.21	32	9	5.85	6.27	13.59
12	54	11	234.2	4.22	16.3
10.33	1.42	5.64	12.39	2.96	237.3
21	5.3	21	3.21	4.53	56.44
17.89	3	11	1.21	2.43	43
219.5	21	1708	1.91	19.64	31
13.4	23.1	4.72	3.58	67.8	43
26.6	21	5.2	3.83	6.75	3.24
16.72	13	13.45	2.25	13.21	10.09
2	25.8	3	7.05	2.23	49.87

8	48.58	43	10.56	6.71	41.73
11	662.7	10.01	12.3	2.43	152.7
32	23	3	2.95	3.21	6.4
4	32.1	12	1.32	1.24	9.01
12	6.51	33	53.85	24.76	63.9
14.71	356	21	3.45	3.21	12
2	4.37	4.25	2.91	4.54	23.5
54	12	11	24.76	7.43	15.09
11	15.84	0.16	27.16	4.5	370
9	6.71	4.98	357.2	3.42	70.53
36	32	5.46	5.26	3.18	21.17
9	15	14	21.27	5.81	16.76
32	6.74	12.07	4.22	5.22	14.14
2	30.92	12.32	4.38	3.09	96.48
12.69	74	32	7.64	5.24	8.14
13	3	119	23.54	2.19	27.18
8.98	32	13	83.22	2.96	149.9
32	5.31	3	5.91	3.27	26.39
21	46.79	11	4.32	2.1	41
11	5.9	3	6.43	2.64	32
11	12.45	12	3.42	3.24	10.68
9	8.87	3	4.53	2.49	10.43
21	32	29.35	2.96	39.79	24.55
69.03	63.82	6.42	3.53	2.31	12
2	11	9	1.21	6.27	2.41
8	436	9.4	24.83	4.45	121.6
12	2	30.03	4.5	2.86	37.07
21	3	43	1.21	4.52	11.22
11	12.36	11	5.14	2.43	22
2	7.85	3.47	2.01	4.45	21.43
9	11	12	10.22	35.65	34.81
11	3.68	43	51.16	23.31	38.71
21	16.48	49	11.29	5.06	370
21	16	3	4.45	6.54	14
3	433.1	45.48	75.21	8.03	46.91
26.62	54	15.87	3.43	3.68	12.18
180.1	12	8.9	4.22	32.3	121.6
33	3	21	23.43	4.5	18.24
78	1205	17	45.6	4.53	21.6
0.63	12.55	21	4.53	6.43	17.07
95.12	7.82	24.6	3.38	8.53	80.12
21	12	7.71	6.54	1.9	8.79
7.16	704.1	43	100.7	1.21	62.6
78.67	8	51	4.16	1.33	29.59
32	125.3	7	45	5.42	18.9
8	3	120	2.64	3.45	32
6.26	9206	12	6.43	5.81	18.94
6.69	172	83	1.21	8.03	6.4
2	6.18	20	7.83	8.48	15.33
221	21	48.44	4.37	500	14.57
9	706	9	5.81	4.76	21.4

4	23	32	5.43	3.42	8.14
4	17.43	11	1.01	3.83	23.5
1.9	52.37	1.9	2.82	2.68	31.43
40.22	41	6.1	9.37	3.44	12
2	2.04	5.03	4.66	1.21	26.6
20.73	3	9.55	1.32	3.54	12.32
21	422.2	3	4.8	23.31	14.51
21	266.8	32	15	2.34	230.6
32	26	33	3.43	3.43	32.1
12	4	170	1.6	3.43	21.21
170.3	9	8	2.84	2.32	43
8.31	12	12	1.4	3.84	10.93
9	21	67	19.43	16.04	3.21
2	29.4	12	24.88	342.1	19.67
8.19	4	134.8	1.34	2.43	184.7
15	9	21	5.24	3.47	14
14	32	15	17.53	34.51	30.98
7	12	103	1.48	2.01	13.59
9	12	6	2.84	4.16	18.34
11	76	15	4.29	1.71	121.5
11	11	11	1.45	6.45	2.41
21		64.66		2.32	
14		21		24.41	
13		54.2		7.69	
8		9.58		2.1	
38.48		37.16		3.05	
5.87		5.97		1.48	
11.29		0.3		4.5	
14		2.5		4.19	
8		32		11.32	
9		14		1.32	
8		6.42		2.43	
9		12		3.83	
43.59		1.9		4.5	
8		3		1.87	
57.88		28.64		2.68	
14.53		56.3		65.3	
4		3		7.89	
21		23		1.34	
22		42		3.23	
14.21		3		2.91	
13.23		25		1.24	
11		9		54.24	
9.06		21		2.32	
11.18		322		1.54	
34.91		21		7.64	
21		21		2.86	
11		7		32.1	
32		4.37		2.1	
14.92		14.92		15.54	
8.06		8		3.43	

12	25.44	2.64
17.64	9.47	3.47
9	22	3.48
17.63	15.17	5.43
11	21.86	1.23
2	7.83	0.93
18.06	31.88	1.23
9	15.35	8.61
9	12	4.19
12	34.07	6.43
21	21	4.8
65.45	13	1.21
9	11	1.21
9	11	1.87
11	3.48	1.6
3.2	23	4.22
62.76	3	3.83
300	4.97	26.86
42.4	34.52	10.75
11.27	12.6	3.67
27.74	168.8	52
11	7.23	5.06
9	22.41	4.25
8.35	12.82	1.88
11	15.32	2.6
11	46.53	1.21
9	6	1.33
11	11	3.24
115	9	4.16
21	26	8.45
11	21	2.52
31	5.26	32.1
29	16	3.81
3	3	2.49
2	12	3.24
21	18.79	8.86
8	6.18	53.28
2	15	2.43
11	34.54	6.27
13.43	4	4.53
7	11	1.6
8	5	4.22
2	3	6.43
34.93	12.96	2.27
2	21	2.31
8	21	21.43
22.4	13	37.3
8	1.87	6.67
12	12.4	4.53
21	11	3.04
9	34	7.43

32	21	24.76
11	5.63	4.57
7.02	21	2.1
11	8	7.11
21	29	0.67
8	12	12.25
9	21	35.65
11.73	12	8.96
21	12	5.89
9	11	16.04
3	7	3.27
9	18	7.64
12	14	2.86
12	16	4.29
2.31	6.29	3.24
9	7.26	2.13
21	32.27	23.06
25.11	39.43	18.05
89	11	4.22
7	5.79	1.32
67	11.94	4.22
18	12	2.13
51	1.21	1.32
3.3	5.47	1.32
12	33	1.54
35.33	10.78	65.3
3.32	8.54	1.55
4.54	5.47	19.21
30.83	8.62	1.53
8	12	41.32
21	41	5.27
9	21	4.58
11.18	32	3.66
21	21	3.54
8	10	5.42
12	41	2.32
103.7	23	1.21
13	49	1.33
33	3	0.67
9	7.98	3.22
8	12	35.65
9	8	1.3
6.35	8	1.71
21	51	24.76
21	21	3.24
9	13	1.3
117	16	8.48
21	11.6	16.04
76	8	45.66
14	21	3.43
20.59	68.36	5.26

11	16.32	4.91
9	12.72	4.34
11	15	1.25
59.6	21	4.22
11.64	12	4.53
1.2	2	1.23
10.77	21.37	4.22
14.5	11	12.71
20.82	0.94	1.88
4	8.43	4.15
11	43	6.7
13.98	9.31	11.33
4.24	12.43	11.6
32	4.67	1.73
32	5.51	23.31
9	43	16.04
23	3	1.54
9	6.21	4.16
6.69	11.1	4.32
14	12	3.43
12	11	3.43
17.72	12	111.21
4	14	1.34
321	4	1.34
11	208	2.95
57.28	12	2.32
14.95	12	3.44
29.54	144.7	4.68
8	9	8.03
21	23	12.25
5	22	3.45
21.35	21	2.32
7.75	10	16.04
7	7.7	5.89
21	10.28	2.84
14.41	0.6	24.13
6	21	4.32
32	32	3.73
8.52	33.17	8.45
9	9.66	4.43
13.38	23	1.32
9	106.3	8.4
8	3	3.83
23.05	11.61	1.23
12	574	4.22
11.82	4.83	1.21
40.01	21	4.22
2	4	2.43
13	21	4.22
8	6.68	2.27
14.23	20.53	2.7

24	14.36	4.53
8	5.8	3.79
12	11.3	4.42
16.62	18.75	4.3
28.71	20.91	6.54
11	12.1	2.59
8	34	23.2
11	14	6.63
21	12	21.32
11	13.43	1.71
11	12	1.32
8.27	7.09	1.91
11	34	5.43
21.2	6	5.64
32	12	5.42
9	8	1.43
22	30	1.21
21	24	4.64
12	3	256.2
21	88	2.32
8	13	5.85
9	6	3.24
65.57	21	1.48
9	9.45	10.37
13.72	852.8	3.27
23.54	12	3.45
1.54	18.38	15.33
21	11	2.64
9	14	2.43
13	15.72	3.67
2	2.96	8.31
4	4	1.21
9	12	4.53
12	12	3.42
32	53	7.54
11	8.39	4.32
9	77.14	1.21
24	12.48	2.1
39.21	10.2	5.63
12	4.32	12.12
4.86	30.6	3.21
21	3	12.31
24.92	7.64	1.43
7.73	21	43.23
11	12	4.32
8	3	2.32
23	197	44.4
9	17.37	2.43
8	13	5.32
4	158	2.43
51.78	16	4.64

20.82	8	2.32
22	8	1.43
8	32	8.96
11	3	2.52
11	21	1.91
32	12	2.43
13	0.6	1.48
21	12	2.31
11	3	16.04
14	11	1.54
11	3	1.3
32	12	2.18
49.58	16.58	4.94
99	13.73	4.2
12	21	1.17
2.34	9	4.22
9	8.01	1.25
8	36.89	1.32
58	12	1.45
66.58	5.55	64.3
15.03	21	1.21
10.57	10.29	2.49
11	3.38	4.84
8.43	10.25	4.64
8	277.4	25.58
21	15.26	20.82
11	10.8	4.64
21	12	10.9
6.66	9.3	7.53
21	23	4.36
23	13	2.71
26.12	10.39	12.23
11	12	4.43
11	12	44.4
24.22	14	5.43
11	21	23.12
25.7	12	1.52
9	11	2.3
21	24	1.24
21	3	1.98
21	8	1.3
24.29	11	3.21
11	21	4.32
11	3	3.43
21	61	2.86
21	6	2.86
2	21	23.43
11	23	4.17
21	21	2.43
64	43	4.17
69	3	234.2

14	9.79	2.86
40.06	32	4.94
21	23	6.43
3.21	6.86	5.89
21	3	8.9
9	7.87	3.31
8.79	8.47	2.95
25.18	21	2.09
22	17.74	3.02
9	6.75	2.94
8.62	2.99	2.5
16.36	18.66	2.38
26.81	9.23	2.91
39.28	3	1.23
11	3	1.45
20.33	6.61	3.42
9	41.25	4.53
126.7	5.98	4.22
7.36	22	3.83
29.48	6.58	2.27
21	10.6	4.63
128	4.56	36.6
8	32	3.43
7.02	4.79	2.56
21	21	1.95
11	4.53	3.42
25.56	25.05	14.42
5	54.68	68.18
9	4.6	1.85
10.55	0.77	1.85
21	32	0.85
8.43	21	10.18
11	9	5.64
11.72	3	2.18
13.98	44	5.89
21	6	2.1
9	5	2.01
9	11	8.03
23	9.73	4.29
38.67	24.81	1.54
7	11	3.48
32	32	2.96
31	16	23.54
9	70.59	4.22
4	37.88	6.43
9	19.64	4.22
21.99	18.4	4.22
51	3	1.25
2	598.44	3.83
9	3	1.32
23	11	6.43

15	11	1.87
6.5	21	26.32
12	5.7	10.86
6.43	5.75	1.19
12	6.6	5.81
2	24.03	3.04
13	21	2.2
8	3	3.87
11	16.64	7.34
12	3.72	2.23
11	99.25	5.74
5	3	5.43
12	32	21.32
9	3.57	6.96
23	78.76	6.71
11	30.08	2.37
21	13.38	3.24
11	11	5.26
9	21	7.4
11	14	7.33
11.57	27	7.35
32.47	11	2.23
14.65	11	7.33
73.03	12	5.26
38.81	3	2.78
121	21	24.76
24.22	23	1.54
32	36	2.01
8	4	5.26
21	18	24.2
9	23	2.18
9.83	15.9	1.53
21	2.15	4.57
21	32	5.81
11	14	1.54
1.32	12	2.32
34	11	5.02
43.74	10.9	3.61
11	13.42	1.43
9	21	8.13
6.51	3.07	2.44
11.24	12	4.19
2	31	1.21
11	21	4.22
11	81.75	1.23
8	12	1.21
21	45.82	1.21
9	191	2.13
14.5	15.89	20.63
19.49	11.78	4.32
9.28	9.33	5.31

23.88	13.36	2.68
31	11.37	18.19
12.2	50.86	1.3
66.41	21.3	61
29.84	3328	5.32
7.05	5.78	3.97
12	3	14.32
277.6	6.48	5.65
8	12	2
11	9.69	4.04
29.54	7	6.54
23	21	3.43
11	1.55	8.96
11	10	4.29
10.15	3	3.27
12.78	16.08	7.64
8	21	2.96
21	21	23.54
8	14	4.16
9	43	4.57
11	10.36	1.45
21	6.72	2.32
9	18.2	2.55
9	23	3.36
9	42	1.21
11	23.52	1.95
11	17.8	2.42
4.45	31.78	2.41
8	12	1.32
7.41	32	6.27
9	13	1.6
0.62	14	2.13
3	2.36	1.87
21	21	1.21
7.19	22.14	2.42
8	31.64	2.25
21	21	2.43
21	5.97	2.75
9	1.65	6.09
12	3	14.32
9	21	17.69
33.82	20.25	2.91
7.75	10.45	3.43
12	7	3.43
9	17	1.34
9	14	2.13
11	32	3.43
7.46	7	4.17
21	7	123.43
21	1	24.76
7.49	101	1.91

9	264	24.2
9	8	2.86
21	3	3.44
11	624	4.16
21	12	2.23
50.84	2	3.79
114.3	1.91	3.47
21	22	2.91
26	9	5.06
21	8	4.01
12	11	4.32
21	9.92	4.57
9	21	3.07
2	18.45	3.24
5.48	7.26	17.68
17.67	13.52	8.95
4	11	1.32
2	7.27	4.53
8	21	2.43
8.61	7.89	4.8
8	9.71	1.21
2.3	3.1	1.21
180.13	2	4.22
45.6	28.64	79
65.57	50.4	7.82
23.4	13.4	10.8
3.11	8.57	3.09
14.38	3.36	2.07
32	4.54	3.54
0.62	61	7.54
12	7.93	7.54
46.09	9.26	2.65
21	12	2.97
12	329.8	1.88
12	3.11	10.77
42	27.13	2.64
0.95	12	10.18
32.1	235.6	5.83
21	8	1.21
20.73	32	3.43
11.33	7	1.24
66.41	32	3.52
8	12	1.33
16.18	1	2.23
21	11	5.06
32	11	4.57
9	54	3.45
9	33	5.81
25.7	13	5.06
89.81	21	1.21
32.87	21	5.81

14.58	21	7.32
21	32	6.43
91.31	10.5	23.13
73	29	7.11
8	12	43.45
9	29.78	2.43
21	6.54	7.44
11	13.32	3.43
12	12	1.25
2	7	21.13
2.17	7.36	3.83
2.31	33	4.5
3	21	1.32
4.84	4.24	2.08
78.67	14.6	2.78
51.78	25.81	7.32
32	14.3	34.8
8	67.77	5.64
2	11.23	4.65
29.76	64.27	4.54
13	43	2.43
9	21	10.3
11	21	4.5
7	5.97	5.25
11	21	4.32
21	7.85	2.32
63.69	3.98	45.6
11	11	3.24
21.99	3	10.18
11	42	0.32
4	21	2.32
16.62	9	1.24
8	31	5.06
21.3	66	0.85
12	3	3.79
11	12	2.01
21	31	1.21
25.08	21	24.76
9	29.42	2.67
38	13	55.33
44	21	2.32
12	29	3.81
3	13	342.1
13	54	2.1
9	27.43	3.21
9	11	3.43
51	9.06	8.61
11	10.2	7.5
9	26.41	4.44
18.28	32	3.12
12	16.36	4.36

13.95	25.5	3.67
13.95	13	4.8
21.99	15	6.43
11	11	1.21
2.1	11	4.22
23.45	32.4	32.4
11.58	9	4.32
2	14.8	4.66
31	0.81	3.54
11	14.82	25.88
12	32	6.13
8.31	6.34	32.32
21	3	7.43
26.83	11.89	26.43
21	12	2.71
32	22	3.43
8	7	1.24
4	21	1.21
35.6	27	1.33
31	9	1.98
30.83	574	5.42
9	88	8.15
23	22	5.26
15	13	1.4
21	30	2.96
12	26	4.01
72.97	27.76	2.13
26.82	228.2	19.71
8.5	3	2.31
9	12.42	1.31
11	21	1.03
21	11	1.23
19	2.21	3.83
2	15.83	2.27
2	5.3	3.62
30.91	10.93	1.77
54.3	6.53	3.43
4	7.51	8.74
16.3	1.71	8
59.42	523.5	4.74
46.88	156.9	25.82
3.43	6.57	6.29
22	5.6	2.01
12	13	6.04
9	9.48	57.43
9	32	5.68
11	54.15	11.16
10.57	186	4.32
9	6	2.32
29.84	21	2.32
14.42	5.93	10.28

8	8.58	0.85
9	33	16.04
21	73	2.32
21	21	1.3
9.94	3	2.5
12	32	4.31
21.71	5.17	4.06
22	14.45	1.56
21	3.81	9.22
9	517.8	6.01
9	3	1.32
18.29	46.83	2.13
12	12	1.87
8	15.78	1.21
14.69	5.35	1.21
21	14	1.45
36.66	1.82	1.23
17.11	7.01	1.62
14.35	28.1	7.14
9	1.4	11.37
15.7	32	9.56
24.2	3	22.45
11.92	21.37	1.95
21	6.25	1.8
9	3	32.12
9	31.3	1.45
21	3	23.43
9	10.27	15.31
9	21	23.1
23	12	1.33
11	54	10.18
12	6	1.21
11.15	21	4.64
9	21	10.77
9	21	1.91
4	11	24.76
32	21	5.89
8	6	2.32
42.36	25.69	3.81
23	2.93	2.96
12	0.6	2.01
11	11	2.86
10.02	12.62	3.41
10.77	6.78	2.66
9.27	6.7	1.99
13.17	3	3.44
11.24	13.44	4.31
7.48	13.96	2.74
8.89	15.26	156.7
35.66	17.44	3.83
0.2	10	4.8

9	33	4.22
9	55.97	1.45
2	21	1.21
21	12	20.68
0.3	11	1.89
39.3	36.64	6.82
21	10.98	2.69
11	14	1.43
8	14.03	1.79
44.05	57.99	2.13
13	23	2.54
31	13.85	1.43
8	11	2.41
21	19.29	7.1
117	4.71	12.25
11	11	2.01
2	22	2.1
4	3.8	0.67
21	3	3.43
44.05	4	1.34
21	12	2.32
16.98	3	3.84
20.25	61	1.4
9	9	2.43
117	12	1.4
33.2	3	43.31
13	3	24.76
21	9	2.18
73	3	5.81
99	326	3.79
34	11	3.43
9	15	16.04
121	12	5.89
9	10.44	9.16
292.34	11	1.21
11.92	9.18	1.32
21	10.59	4.5
7	11	3.83
21.88	3	4.53
32	12.81	14.95
20.25	10.43	3.43
9.8	43.32	35.7
57.28	11.15	4.92
6.85	27.53	12.43
32	12	8.96
22	33.26	8.89
12.55	13	5.81
21	23.27	2.91
9	14.97	4.29
8	29.46	2.86
9	10.17	82.5

11	3.01	9.37
9	7.83	2.88
9.34	97	3.43
21	93.21	1.3
6.46	21	1.48
23	11	2.64
5	11	1.71
6	22	1.21
9	3	2.1
15.3	18	1.48
21	60.87	2.64
22	50.57	9.97
31.5	12	4.24
8	98	2.18
9	21	24.76
8.43	3	4.29
8.67	8.41	2.23
59.51	25.24	147.2
9	3	23.06
22.78	12.06	2.17
2.5	13.66	8.89
21	60	8.31
2	9	1.23
2	1.44	22.34
9	12.78	2.43
1.35	3.8	4.8
8	12	3.83
12	5.32	1.21
20.28	2.43	1.45
4.69	4.69	1.87
9	9.82	2.2
1.36	11	1.2
12.1	18.29	4.22
161.8	12.6	4.92
21	17.99	15.58
11	3.42	2.32
8	1.98	38.29
33	30.51	23.11
21	23	14.43
21	3	4.01
14.26	32	2.96
11	29	1.24
11.92	145	3.81
11	32	16.04
10.32	8	23.54
21	574	1.4
11	5	1.48
222.2	12	3.43
89.81	21	8.15
32	44.16	3.21
21	166.5	3.81

34.04	0.6	4.21
12	21	1.21
11	33	2.23
33	13	8.15
21	12	5.33
12	4.54	39.22
21	21	12.56
9	33	1.82
11	7.43	3.55
21	32.96	1.21
22	2	2.43
32	44.47	2.32
4	54	5.65
32.23	98.01	4.65
12	8.49	15.67
21	13	5.43
21	13	10.22
71.2	30	1.48
32	54	1.4
15.95	8.18	4.01
43	19.87	1.4
61.81	22	34.51
21	32	5.29
7.59	17.47	1.32
8.62	59.19	11.16
11	12	3.21
2	18.7	6.43
8	14	1.21
349.11	2460.09	1.32
11.09	25	152.5
45.57	61.52	4.73
43	13.73	2.77
58.73	14.23	10.38
32.49	4.32	44.43
7.78	12	4.17
21	12	3.81
21	32	3.79
2	11	1.24
21	12	2.64
2	43	4.32
11	21	1.54
23	9.54	3.43
4.22	71	4.32
8	15	2.32
14.45	3	5.73
12	9.81	8.92
4	3	1.2
9	5.26	1.23
26	3	1.87
2	29	6.43
1.6	14.31	4.22

21	15.14	2.27
15.63	11	33
8	9.59	6.27
11.12	13.23	2.14
12	5.43	2.58
8	90.75	33.54
8.98	114.8	3.43
11.64	11.04	35.96
21	12	6.08
23	3.69	41.21
21	24	3.43
10.03	21	2.01
23	11	16.04
9.92	12	1.4
11	57	1.71
8	23.38	4.01
130.7	25.03	2.32
25	3	4.01
21	12	3.24
13	3	5.26
35.63	7.71	6.1
12	14.56	8.13
8.18	7.53	3.86
17	17	1.6
4.97	14	4.8
13	11	1.21
4.29	3	4.53
23	11	3.42
2	574	1.6
12	11	3.42
11	15.23	3.84
8	17.81	35.99
8	3	52.2
11	12	5.64
13	13	5.84
21.07	21	4.52
11	12	10.74
32	21	0.43
21	4.1	2.03
63.57	6.29	342.1
4.24	23	8.64
11.12	11	3.52
21	15	9.37
11	21	8.45
8.64	21	6.54
54.03	3	5.89
21	32	4.57
6.69	7	4.16
8	13	5.64
1.28	306	6.35
137	11	9.97

6	143	5.33
43	23	2.1
31	3	48.46
8	38.52	23.41
1	33	3.83
2	5.87	4.5
21	2.18	2.04
12	12.59	4.53
12	14	2.6
11	21	4.34
12	21	5.83
22	8	1.43
9	12	3.43
9	32	1.98
8.27	43	5.83
10.83	18	2.78
9	11	23.31
21	28.43	3.45
13	52.68	5.26
87	3	2.52
33	1.34	4.16
9	18.28	81.24
32	270	4.01
32	4.22	100.6
33	7	1.78
18	10	1.21
32	26.07	36.54
10.83	22.96	3.89
32	7.1	5.85
11	4.53	5.19
2	12.1	4.13
13.23	11.48	27.61
6	197.6	17.69
14.71	16.78	3.73
2	16.08	8.48
5.65	3	2.1
11	21	4.54
11.66	19.26	1.3
10.55	39	3.21
11	12	5.85
9	12	4.57
13.11	21	7.35
8	3	3.21
13	26.65	5.81
1.77	21	5.33
9	3	3.45
11	7.18	23.43
2.3	7.91	7.99
5.02	67.45	2.31
8	11	1.23
9	21	3.42

9	10.94	1.45
63.23	11.45	6.43
7	31	4.5
7.36	7.36	4.8
12.35	121.9	1.23
4.86	20.39	1.24
22	6.46	48.46
11.33	12.93	3.43
8.94	9.64	1.69
9	31	1.51
13.11	12	2.82
12	8	3.27
11	12	10.22
11	31	7.35
87.24	3	24.76
9	0.6	3.45
11	6.18	2.01
9	11	2.1
9	13.69	3.83
8	11	1.32
31	9.61	27.23
11.82	407.7	86.5
12.98	9.89	1.09
7.54	6.44	3.12
4	23.21	2.32
6	4.72	2.64
21.35	6.14	234.2
21.07	21	4.16
9	62	2.91
7.41	12	1.21
21	91	2.32
14	9	3.21
12	31	1.87
8	6.3	1.23
4	8.78	2.21
12	10.91	5.18
11	23.04	37.63
4	3	1.21
9	37.88	8.96
3.11	14	5.24
11	21	4.17
21	9	3.27
11	11	3.24
3	3	4.66
16.02	17.17	1.87
7	12	6.43
2.32	40.6	1.45
21	3	342.1
23.27	21	2.01
9.39	8.87	28.46
14	15	1.25

11	6.16	3.43
13.09	112.3	1.32
9	35.06	1.32
2	11	1.16
24.49	27.41	7.48
9	21	36.08
33.88	10.02	3.81
11	12	4.63
22	3	2.55
55.86	29.29	36.82
7	6.58	2.31
7.41	9.12	5.17
2.33	35.52	28.61
1	21.29	1.32
8.86	9.97	34.2
11	21	4.53
19.38	21	3.81
11	21	3.84
9	32	2.32
8	10	2.01
2	21	5.81
22	4	10.77
65	18632	4.22
11	12	16.04
14	11.42	3.37
99	12	1.32
23	13	10.18
14.95	4.65	40.13
12.78	11	23.43
43	7.58	23.42
23.27	10.62	4.32
12	21	4.8
4	6.6	2.31
11	31	1.23
11	24	5.89
2	11	2.01
17.83	5.17	16.04
21	21.05	1.32
21	11	2.27
2.1	11	1.32
9	12	2.32
11	23	1.21
27.83	69.03	1.43
32.1	6.8	2.17
4.78	12.46	6.2
12	12	3.43
7.05	4	234.2
9	9	1.24
64	7	3.81
81	12	35.65
2	14.78	1.34

11	21	1.25
21	15.03	1.45
12	147	6.43
321	3	11.73
21	34	1.24
12.31	33	5.81
10.46	4.23	3.47
121.8	21	6.43
4	3	4.19
13	83	3.43
11	21	7.54
21	16	4.16
11	13	3.45
21	6	0.85
9.94	11	2.32
43	122	14.24
2	3	1.45
9	32	5.43
8	3	342.1
9	20.35	1.7
11	6.44	27.75
8	10.6	1.93
76.88	5125	5.13
31	13	1.43
21	21	342.1
2	12	342.1
11.28	11	2.27
11	1.3	3.53
5.48	22	2.32
9	29	2.01
9	12	35.65
2	24	8.45
20.62	160.8	1.48
86.14	2.69	4.5
59.65	11	8.45
21	13	4.16
11	8	4.57
11	11	3.73
75.34	8.43	12.99
33.5	4.6	4.85
6.67	25	6.43
2	9	31.21
12	10.21	0.81
7.4	31.48	32.1
103.7	834.9	5.43
20.07	19.1	5.64
23	10.74	52.93
38.81	14	14.54
21	12	7.76
31	32	123.43
4.84	5	0.32

11	8	2.32
32	32	4.16
9	12	7.64
53.05	12	3.79
11	141	3.21
10.81	249	2.1
9	31	3.81
4	3	1.32
55.48	545.5	53.4
11	12	12.42
53.05	100	16.06
23.02	16.04	16.42
9	5.66	4.33
11.1	12	5.53
11	8	7.33
21	3	4.01
11	3	2.91
25.67	10.3	2.18
12	0.6	1.4
12	23.15	8.45
15.95	12	6.45
23	7.8	2.22
86.37	11.52	31.15
23.52	6168	4.33
2	11	1.21
16	21	3.42
31	3.21	4.53
1.91	11	1.21
15.28	52.6	10.15
61.25	12.43	65.3
6.46	14	32.4
7.76	18.8	3.21
5	12	67.3
13	3	3.73
32.23	17	3.24
20.07	2	1.21
32	5	1.3
12	28.86	6.43
12	24.42	2.72
10	13	3.83
31	12	2.13
20.67	7.22	4.53
37.43	26.37	2.02
11	5.64	1.99
4.87	41	32.2
11	12	3.31
12	10.11	23.84
3.21	12	3.24
4	701	4.22
6	1	123.43
104.2	14.32	5.65

13	6.43	3.42
13.95	12	1.43
21	21	2.96
9.85	32	3.21
32	25.91	24.76
9	13.22	8.45
21	22.2	4.16
19	21	4.32
2	11	1.83
11.49	28.08	3.65
43	3	4.5
15.53	11.2	1.78
6.71	9.51	3.83
9	11	1.23
73.73	8.61	1.32
11.58	12	9.86
9	1.76	1.93
31	17.27	6.32
10.32	53.82	13.32
73.03	5.48	12.31
81.56	34.86	7.33
21	12	1.54
9	20	5.81
7	21	3.81
9	21	23.43
4	18	2.32
2	12	2.43
22	3	1.6
7	12	1.21
8.34	8.15	2.14
35.6	236.1	3.44
11.72	5.24	21.43
32.47	34.71	32.4
71.2	198	12.48
32	12.9	6.54
21	21	35.65
3.43	61	7.54
8	15	1.33
16.56	14.92	223.43
11	29	2.43
9	7	8.45
31	10	5.26
13	12	2.32
19.86	24.17	4.21
9	3	1.23
9.06	344.4	16.12
9	12	12.43
54.03	661.9	16.04
2	41.15	23.54
24.92	3	5.06
21	11	4.29

11	3	12.25
9	3	2.49
11	48	5.89
8	17.19	3.51
21	12.44	3.48
2	27.03	1.23
12	12.22	1.21
7	10.52	4.14
12	3	56.2
23	228.6	1.48
6.43	54	1.3
32	3	123.43
26.81	21	3.44
4	3	2.91
19.47	30.66	2.32
10.1	3	5.81
21	3	1.3
22	22	2.64
21	32	3.43
11	3.56	3.43
13.64	13.48	1.54
9	9.05	2.32
12	21.42	1.56
14.69	11.96	4.4
21	22	1.32
8	15	4.8
24.29	14.6	4.56
13	115.5	9.01
12	1.54	5.75
16.98	21	1.21
21	3	1.3
99	43.92	34.21
13.01	8.59	13.51
26.28	9.24	2.45
11	11	4.5
60.85	26	1.32
22	2	4.8
8	1.54	13.45
21	3	15.37
32	10.91	1.4
12.79	10.48	4.64
161.8	22	2.32
8	12	8.48
12	8	5.89
8	75	23.54
11	11	2.91
98.79	5.5	2.23
13	22.4	4.29
43	50.66	2.32
3	13	4.01
103	12	4.29

21	21	2.23
11	33	3.1
8	22	1.29
12	19.52	4.62
163.1	11	6.11
9	27.23	4.8
9	34	12.4
7.46	17.3	1.26
21	12	2.36
21	3	4.32
8.19	30.45	65.3
11	15.89	3.27
31	21	1.43
11	181	3.21
2.31	7.34	39.98
16.97	245	5.01
14	11	2.01
16.02	3	3.42
11	6.94	4.94
8.45	8.51	43.21
9	14	3.23
95.12	41	3.43
4.63	8	1.43
136	12	6.27
30.01	32.52	8.54
9	19	4.19
8	47.43	4.53
9	2	4.53
21	29.21	1.23
8.64	14	4.78
21	3.87	25.73
200.2	76.42	1.23
9	21	4.57
9	6	16.04
9	31	3.83
16	21	8.61
135	21	2.13
3	3	2.27
8	1.34	5.32
11	13	24.42
9	21	11.56
83.22	85.91	5.42
9	602.2	23.54
21	13.77	23.43
14	19	9.34
21	27.15	2.63
19.4	26.32	8.54
5.58	4.47	26.92
2	2.35	1.81
9.11	13.99	5.32
21	26.59	1.91

8	66	3.54
9	12	2.32
22.84	18.43	7.35
7.94	11	3.45
23.88	53	2.95
41	9	10.18
11.27	21	4.54
7.13	11	8.03
6.29	6.6	4.57
9	3	1.54
2.34	11	4.8
65.3	12.78	34.95
3.43	351.58	4.22
7.16	15.13	2.35
4	21.61	3.75
21	14	4.21
12	12	32.1
21	14.6	4.26
21	3	2.23
14.41	15.66	6.27
9	31	1.43
18.42	11.45	17.54
11	17.41	42.19
11	2.14	3.73
22	56	5.24
26.83	19	3.27
8.45	12	5.64
9	5	4.32
9	46.08	3.17
77.2	3.46	20.47
11	660.7	1.23
10	12	2.27
9	34.54	2.27
4	3.31	10.64
12	9	1.21
200.2	10	23.43
65	471	2.64
14.54	16.31	6.27
32	65	342.1
22.64	8.84	39.98
4	33	2.13
36.67	10.66	2.43
21	21	2.78
11	12	3.45
23	12	2.43
90	6	1.54
11.43	3	1.87
8	3	4.8
87.3	12.14	2.32
24.29	21.12	2.89
4.95	9.52	1.32

105	20.79	4.53
11	8.51	2.35
27.43	21.06	5.25
8	32	2.08
8.04	21	3.73
8	32	1.43
9	32	2.32
9	14	7.64
23	11	2.91
3.17	7	2.32
117	14	2.78
7.92	26.87	17.83
9	5.51	7.76
9	1.81	2.7
4	23.91	6.27
4	2.5	3.83
2	22.13	2.36
19.49	22	4.64
17.89	12	10.77
43	0.6	2.49
26.48	21	2.32
11	269	2.32
21	3	7.54
32	8.86	4.87
21	3.84	13.3
32	3.9	1.02
4	12	3.43
8	11	3.43
25.56	73	3.43
89.81	12	2.1
16	59.61	3.43
4.22	22.87	34.51
9	3	2.23
9	11	1.6
8	11	2.27
72	39	1.23
13.25	18.2	4.22
54	3.82	7.64
9	6.08	5.68
21	41	1.01
32	3	10.74
21	267	5.81
15.71	6	2.32
12	8.67	1.32
222.2	139.6	2.65
144.72	256.4	42.1
40.71	43	2.18
9	23	23.43
11	23	2.49
17.4	6.45	4.31
21	1.23	6.32

21	574	2.13
37.87	1088.68	6.43
121	60.52	7.16
21	3	23.54
2	11	4.16
10.41	7.27	1.72
9	3.52	9.87
9.8	21	11.23
21	21	4.22
9	12	1.32
18.04	22.54	3.48
3	76	1.32
9.86	13	1.32
21	12.48	3.84
9	23.64	5.81
13.74	22	3.45
30.81	2.39	2.01
35	34	23.31
80	11	2.27
58.73	117.4	4.23
21	12	2.32
4.59	12.19	6.43
89.81	12	123.4
5.75	46.5	2.65
9	7	1.21
21	27.52	2.41
2	665.34	3.42
5.43	16.45	7.11
9	11	4.29
7	251	13.43
110	9	24.76
8.53	12.16	1.7
29.09	15.61	6.43
7	12	67.86
9	8.47	8.03
9	24	3.81
7.75	12	256.2
115	237.7	3.21
22.81	14.52	3.81
12.64	11.24	1.95
14	54.25	4
21	3	23.31
9	12	4.32
6.33	8.5	39.84
39.88	9.59	6.82
41.43	12	446.2

μg/L)		TPSA (ng/L)	
NLM		LM	NLM
18.8		0.87	5.6
15		1.22	1.32
23.25		2.42	0.34
57.89		0.26	0.86
12		0.25	0.26
6		1.2	0.15
10.29		0.97	0.87
22.5		2.12	1.54
17.25		1.32	1.2
8.34		1.54	1.87
39.16		1.43	0.87
24.38		1.98	1.22
14.59		0.86	0.39
21		0.7	2.32
38.05		1.43	0.37
14.49		1.53	0.54
14.87		5.3	76
10.69		0.85	1.54
21.83		0.54	6.3
16.77		2.54	0.87
12.09		7.8	0.87
6.36		5.64	0.66
13.59		0.87	1.06
370		3.21	5.3
4.3		0.34	1.53
14.41		1.3	7.4
9		2.42	1.98
23.5		0.86	1.98
41.06		1.34	1.64
12.33		1.3	0.98
11.36		0.97	0.54
11.03		0.98	0.87
26.01		0.96	1
16.06		1.87	0.46
23.1		1.32	2.7
13.81		1.75	1.3
18		1.3	2.56
21.17		1.17	0.98
2.41		0.79	0.87
9.01		2.26	1.43
12.93		1.43	1.34
21.05		0.76	1.47
11.02		0.26	0.87
32.38		0.97	0.89
17		0.26	2.32
13.51		1.2	0.78
12.26		0.65	2.32
12		0.66	0.53
17.3		3.2	1.3

184.7	0.65	5.6
24.13	1.54	1.14
10.13	2.7	1.53
3.21	2.32	1.54
14.46	1.98	0.98
58.2	0.21	6.3
23.85	0.56	0.48
21	1.43	1.3
14.7	0.9	0.76
16.03	0.89	0.54
14	0.46	0.54
16.47	0.99	0.86
17.07	0.64	1.98
43	0.43	1.54
6.36	1.23	1.98
23.46	0.12	0.98
22	1.3	2.32
27.55	2.32	1.2
78	0.98	1.06
25.76	1.2	1.25
14.41	1.26	1.3
21.83	0.58	0.87
25.71	1.32	1.45
34	0.97	2.54
21.2	1.65	1.43
32.02	5.3	1.3
10.87	0.93	3.5
18.4	1.32	0.98
23	0.76	3.4
8.14	3.5	0.96
19.2	1.21	1.21
31.08	2.43	0.98
14	0.23	1.53
14.01	2.12	1.2
21	1.54	1.76
9	0.65	1.23
14	1.26	1.98
16.87	1.23	0.54
21.53	0.97	1.54
12.4	1.98	1.54
15.74	1.54	0.76
78	0.99	0.66
11.42	2.31	0.87
30.89	0.54	0.09
32.22	1.35	0.54
12	1.76	0.9
27.79	1.98	7.4
17.8	0.94	5.3
20.83	1.53	0.34
12	2.42	1.3
17.07	0.98	1.54

12.27	1.67	1.75
12.35	0.41	1.4
12	0.33	1.98
51.64	0.54	0.87
18.24	1.03	0.97
38.44	1.32	1.34
68.27	0.78	0.68
2.1	0.86	1.43
6.55	7.4	1.54
20.95	0.65	1.45
13.15	1.86	3.56
11.64	1.2	0.54
257.1	0.98	0.43
10.58	0.65	0.94
32	1.98	0.54
10.92	1.43	0.79
15.18	1.32	2.99
21	0.76	0.26
19.65	1	1.54
18	1.98	1.23
10.7	7.8	1.65
13.53	7.4	0.76
47.67	1.22	0.87
22.29	5.3	2.08
32.85	0.97	1.2
205.7	1.75	0.4
105.4	7.4	1.03
32	2.99	0.23
8	0.21	0.26
11.07	0.54	1.54
16.76	0.87	0.34
3.21	0.23	1.32
27.94	7.15	1.56
21	0.78	0.99
23.1	5.67	1.76
12.81	1.3	1.01
43	0.79	3.54
11.64	0.46	1.54
16.77	7.4	1.4
12	1.76	0.46
16.87	3.83	1.45
11.59	1.76	0.85
14	0.57	1.53
12	1.98	2.1
6.4	5.6	5.5
32	1.43	1.98
25.63	1.2	1.54
21.63	0.87	0.54
21	0.87	0.22
96.74	1.3	2.32
9.47	2.32	1.67

16.47	1.65	0.98
12.8	12.3	0.99
15.05	1.36	0.35
22.8	0.43	0.09
49.88	0.18	1.23
14	2.1	5.3
2.34	1.76	0.86
9.07	5.56	2.1
15	1.54	1.06
9	0.87	0.87
19.65	0.99	1.76
16.87	1.54	0.56
21	0.54	1.57
23.1	2.3	1.87
12.43	2.99	1.34
32.67	1.3	2.1
11.64	0.71	0.33
23	0.86	0.7
6.55	1.54	0.86
14.87	0.43	1.54
15.8	0.56	1.65
26.68		0.69
18.22		1.87
41.51		1.67
25.07		2.32
18.51		1.32
22.76		1.23
4.88		0.39
32		0.86
22.6		0.34
20.95		0.61
17.29		0.26
23.6		1.98
8.79		0.26
32.32		0.99
20.38		2.08
15.22		2.4
145.5		0.35
9		1.2
11.7		2.1
52		0.12
12		1.3
184.7		0.45
12.18		0.26
22.65		1.54
21.17		0.54
9		1.56
13.4		1.23
11.23		0.76
22.56		1.3
15.6		1.75

12.58	1.23
12.33	0.67
13.36	0.65
52.57	0.98
32.12	0.31
53.01	1.24
110	1.43
21	0.97
5.64	5.6
29.59	0.72
21	1.68
8.79	1.2
17.29	5.6
14	0.54
23	0.54
39.16	1.34
14.14	0.87
73.9	0.36
16.3	3.56
9.63	2.24
17.79	5.32
34.96	0.26
24	1
9.32	1.3
12.55	1.53
16	1.53
34.96	0.87
14	1.06
184.7	0.54
39.16	1.87
1.32	1.65
23.17	0.59
15.65	1.3
41	0.87
70.46	1.98
46.55	1.43
27.16	1.32
21	0.9
12	0.54
8.14	1.22
16.76	1.3
8.14	1.4
23.1	0.97
11.42	2.32
39.16	0.54
22.6	0.99
32	5.3
41.88	0.34
12.82	7.4
12	2.7
12	6.3

40.59	0.61
15.18	0.99
12.68	2.32
9	1.53
23.72	0.26
49.87	1.78
48.29	0.87
32.1	1.54
15.82	2.31
21	7.8
32	0.9
10.63	0.86
23.1	2.99
4.53	0.54
123	1.76
101.4	0.65
46.74	1.54
39.37	1.23
23.2	0.99
19.23	0.98
22	0.54
31	2.32
2.43	3.2
32.22	2.99
13	0.87
32	2.76
11.76	3.56
10.66	0.87
12	1.54
22.65	1.86
191.5	2.43
9.01	1.32
21.94	0.5
15	5.6
11.76	0.66
17.37	0.99
15.52	0.61
11	1.68
18	1.76
12.9	0.52
370	1.32
12	1.75
15.6	0.54
15.5	1.3
14	1.98
10.69	1.34
370	3.3
28.34	2.12
31.15	0.97
13.39	0.98
58.35	0.41

9.37	1.98
18.3	1.23
6.55	1.4
12.8	2.31
21.32	2.7
21	6.3
21	0.99
13.39	2.31
9.56	0.73
11.82	0.34
10.63	1.75
14.4	0.87
13.53	0.75
9.22	1.75
14	0.87
18.11	0.98
23	0.54
7.7	0.87
13	0.54
5.5	1.3
18.34	0.87
14	0.46
14.95	0.86
15.52	0.46
7.95	0.54
31	1.54
12	1.98
42.3	0.33
32	1.23
23.6	1.54
16.76	0.26
17.46	0.34
12	1.28
26.17	2.12
16.6	1.43
13.62	1.98
107.6	0.97
10.69	2.99
21.57	1.43
370	1.58
21.1	0.28
17.8	1.34
10.67	2.42
27.5	1.4
4	0.26
22.53	0.54
23.43	0.72
19.01	0.87
18.24	1.54
17	1.56
28.44	0.89

145.5	0.34
11.76	0.99
11.66	3.5
107.6	3.5
16.87	0.32
12	1.23
6.36	1.32
43.61	0.97
12	1.75
20.79	0.46
14	1.43
60.13	1.35
14.41	0.26
12	2.31
12	1.98
15.52	0.7
9	0.26
15.82	1.06
107.6	1.23
12	0.87
39.16	0.56
6.55	0.98
17	1.65
15.35	1.78
15.13	0.54
23.4	0.54
15.98	1.19
16.77	1.3
8.79	1.98
25.4	0.87
14.98	0.98
14.87	1.2
48.29	7.4
21.17	7.4
11.22	0.46
13.99	1.65
21.16	0.53
21.4	0.41
22	1.16
22	2.7
17	1.65
13.17	2.26
17.4	1.53
11.51	1.2
21	0.87
15	1.2
14	1.22
21.2	0.79
14	1.76
12.62	3.2
14	0.786

52	0.87
13.93	0.66
102.2	1.43
23.1	0.26
9	0.99
12	1.34
11.9	1.45
14	0.45
12	5.5
29.59	0.87
12	5.3
15	2.99
24.99	0.87
34.11	0.65
60.12	0.67
23.85	2.1
1.32	2.56
23.85	1.54
27.19	0.87
37.5	6.94
66.77	1.3
13.36	0.35
58.57	3.21
6.74	1.86
22.12	1.54
9.82	0.5
10.93	4.6
19	0.7
43	7.4
22.06	7.8
12.85	1.65
21	1.75
12	2.12
16.12	1.54
27.68	0.97
21	0.99
13.57	1.54
11	0.43
32	3.2
17.37	1.98
15.52	7.4
25.35	1.53
4.53	0.7
10.7	0.26
34.96	1.23
34.96	1.98
21	1.65
41	0.54
16.76	1.967
28.57	1.43
30.89	0.54

23.38	0.89
19.52	0.34
12	0.786
20.64	1.24
17.42	0.87
11	1.25
20.96	0.87
7.67	0.93
9.9	0.41
3.96	1.23
13.4	0.84
7.15	2.12
12.68	1.06
271.9	1.2
39.16	6.3
21.83	0.66
11.36	1.45
9.12	0.97
121.6	0.98
32	0.61
17.44	1.78
15.8	2.7
17.07	1.06
72.96	0.43
8.59	0.75
43	3.21
21.2	0.48
22.5	1.2
10.5	0.97
14	3.2
31	0.54
11	0.9
18.34	1.43
14	1.75
20.46	1.53
78	0.72
14.87	1.76
12.93	0.26
20.14	1.98
65.97	2.12
12.3	1.3
17.07	2.7
23.1	0.87
15.28	1.4
12.37	1.2
15.36	1.53
12	0.66
14.32	0.87
12	0.98
21	0.89
60.13	0.9

12.4	1.54
17	5.6
15.84	0.77
32.8	0.76
17.46	0.94
9.01	4.6
14	5.4
21	3.5
23.6	0.97
20.64	1.76
8.46	1.75
8.7	1.53
14	0.43
40.23	1.53
21	1.53
26.36	1.22
22.16	2.01
11.81	1.54
13.94	0.61
21	1.3
21	1.3
10.63	0.76
23.1	7.8
9.35	0.99
17.29	1.76
12.1	1.54
52.67	0.87
13.93	0.58
10.93	0.54
101.6	0.43
11.36	1.89
14.14	0.1
250.2	0.97
27.19	0.54
32.1	1.23
62.85	5.12
15.15	0.87
21.1	0.87
21.32	1.56
23.27	1.32
17.12	1.32
18.33	0.87
3	0.61
6.55	0.33
13.43	0.87
21.32	0.65
107.6	0.97
17	0.87
39.45	3.5
21	0.43
13.45	0.23

11.51	0.94
78	3.56
13.86	0.53
32.22	0.65
21	4.6
17.29	1.43
11.44	1.43
21	1.32
9.7	1.32
11.78	7.4
12.09	3.5
19.01	1.43
19	2.31
13.43	1.34
16.7	1.32
9	0.41
13.43	1.76
4.53	0.26
8.14	1.54
31.05	1.23
12.84	0.78
13.81	0.78
35.15	3.36
39.93	1.67
9.21	1.76
9.7	0.67
26.16	0.41
11.88	1.35
22.59	0.33
370	0.66
3.21	2.32
23.1	1.54
10.68	0.87
11.64	2.7
9.52	1.85
28.02	1.26
12	3.5
16.04	0.94
21.76	0.94
4.12	1.43
9.39	1.75
29.43	1.9
12	1.98
12.1	1.32
13.81	0.98
22.6	1.34
38.44	0.54
49.88	1.54
12	2.13
12.8	1.2
23.2	0.99

1.32	0.56
23.1	0.99
16.47	0.79
184.7	0.51
23.2	1.32
13.43	1.34
13.4	2.91
32	0.87
12.4	76
41	2.54
12.99	1.76
77.01	1.16
24.8	0.31
24.62	1.43
24.82	0.51
21.49	0.78
63.9	2.32
1.6	0.87
2.41	0.87
12	0.26
43	0.99
52.55	0.46
12	76
14.71	2.7
9.6	0.15
21.79	3.56
12.68	0.76
23.15	0.65
32	0.87
32	0.23
18.85	0.75
12.4	5.6
11	1.75
7.95	1.43
18.34	2.56
13.64	0.87
41	0.43
18.92	0.87
43	0.87
18.6	0.66
18.6	0.61
10.68	0.43
12.3	1.3
32	1.76
8.3	1.76
11	0.54
14.95	2.32
49.88	0.54
12.8	1.32
12	1.43
78.95	1.32

78	1.2
9.02	1.54
16.32	0.97
13.43	0.65
23.43	1.67
21.3	1.76
12.71	0.78
61.01	0.49
48.29	1.32
23.2	2.54
21	2.56
32	0.7
13.81	0.98
17.96	0.52
12	0.65
54	0.86
17.8	0.99
31.54	0.7
9.91	0.53
23	5.6
31	2.7
22.53	1.75
15.42	0.43
16.09	1.4
9.35	1.75
4.64	0.26
54	5.7
21	0.87
8.27	0.52
13.1	1.2
34.96	0.54
15.82	0.54
32	1.89
10.93	1.43
17	0.26
25.35	2.32
51.04	0.54
21.4	0.26
31.94	0.29
370	3.56
12.8	0.56
17.25	0.46
20.95	0.61
17.29	0.26
37.75	1.3
21.09	0.76
12.52	0.67
11.16	0.65
12.71	2.43
148	0.65
44.18	2.32

10.89	1.43
38.61	1.43
14.54	1.75
1.32	3.2
49.87	0.66
76.3	1.54
7.6	3.5
30.42	0.3
13.43	0.46
15.65	2.7
11.36	3.56
2.41	4.3
4.2	1.54
13	0.67
14.84	0.87
32	0.99
7.95	0.56
15.82	0.87
9	76
13.81	0.54
9.01	1.43
48.29	1.17
11.78	2.32
16.47	1.98
15	0.43
11.32	1.43
32.13	0.67
35.3	0.79
12.54	0.65
33.21	1.89
11.7	1.45
14.41	1.3
24.55	1.98
16.87	1.54
41.77	0.87
21.67	0.75
3.067	2.6
16.12	1.16
21.64	1.54
15.2	2.42
4.48	0.87
15.44	0.43
11.9	0.21
15.39	0.43
12.4	1.32
20.3	1.4
8.98	1.53
11.2	1.22
9	0.9
12	1.43
18.4	0.55

28.57	1.32
8.43	0.89
49.88	5.3
48.29	0.46
19.8	0.87
11.42	0.79
19.28	3.36
2.12	0.98
12.06	0.03
17.57	2.35
14.54	1.32
12.32	0.46
7.73	2.99
101.6	1.98
15.18	0.54
13.43	1.75
12	1.54
11.34	1.21
16.07	0.45
17.77	0.21
14.14	7.4
17.25	1.43
22.6	0.86
18.82	1.54
14.5	1.3
12	1.53
6.23	3.2
17.46	1.3
12	2.32
41	3.56
14.84	5.6
12	0.87
12.18	0.54
14	1.65
121.6	1.98
23.85	0.46
15.82	1.43
11.22	1.34
17.08	1.98
36.58	2.32
25.88	0.78
7.73	2.56
14.96	0.37
49.56	1.34
32.12	0.34
15.98	0.09
55.64	1.54
18.74	1.56
24.55	1.12
22.06	0.86
16.77	0.7

17.25	0.54
32	1.3
17.29	2.56
12.89	1.43
17.29	1.68
12.43	2.54
13.16	0.49
32.1	0.67
17.3	0.87
21	7.4
7.73	7.4
12.8	2.1
32	3.21
14.12	1.4
51.64	0.45
11.52	0.54
21	1.3
13.4	1.54
11.78	0.87
14	0.98
32.22	1.2
23	2.99
8.24	0.54
18.6	2.32
8.24	1.54
37.78	0.94
14	3.5
49.87	5.3
25.35	0.45
370	0.98
14.41	0.87
16.77	0.98
21	1.06
13.31	0.72
9.01	0.99
12	1.43
4.53	1.2
11.2	1.53
11.42	2.01
19.65	0.33
29.07	0.87
23	7.4
25.35	1.02
10.12	0.88
13.51	0.86
29.59	1.75
13.81	0.98
35.8	0.87
13.31	0.26
47.49	1.23
100.8	2.99

17.5	1.54
14.01	1.43
41	1.98
19.2	2.12
21	0.97
13.59	0.87
23.2	0.99
8.14	0.87
19.65	2.32
12	1.98
18.79	0.56
32.67	0.76
17.21	1.43
23.6	0.54
12.4	1.43
19.49	0.95
15.27	0.78
46.72	0.76
43.9	0.65
5.26	0.89
26.13	3.36
13.34	1.32
22.12	0.76
24.31	0.87
32	0.54
43.39	0.86
21.17	1.42
12.32	1.75
9.76	2.1
15.65	0.7
2.41	0.79
8.79	0.46
12.4	0.46
11.58	0.76
370	2
15.43	0.43
32	2.32
32	0.87
4.53	1.54
6.85	0.86
10.47	0.54
11.22	1.76
7.73	2.4
32	1.3
12	2.32
43	1.2
14	1.43
32	1.98
11.2	1.45
93.74	0.26
61.82	3.3

12.07	0.45
23	0.56
12	1.54
12	0.46
14.7	2.34
38.88	0.4
32.1	0.35
3.51	1.21
20.52	1.32
23.85	1.53
21	0.86
23.97	0.46
43	0.65
10.62	2.32
39.16	1.54
18	5.3
10.68	1.43
14	1.43
14.41	1.87
16.51	0.09
22.06	0.56
23	1.75
23.13	1.67
14.55	0.4
22.75	0.29
23.13	0.93
17.34	1.43
14.82	0.87
12.09	1.23
3.85	0.66
20.46	1.36
55.72	1.77
20.58	1.32
13.37	1.54
43.77	0.98
8.14	0.87
23	1.32
19.65	2.01
15.18	1.98
20.46	1.43
32	2.13
26.49	0.89
32.1	1.83
23.63	1.4
18.89	0.69
45.23	0.98
21	2.32
9.01	0.26
14.41	0.85
22.6	2.7
22.6	1.59

9	0.34
18.9	6.3
271.9	1.21
9.6	0.1
27.25	0.65
14.73	1.2
10.57	1.56
13	1.75
21	1.2
13	3.5
18.6	5.3
41.77	1.54
23	1.43
11.33	1.43
21	1.21
28.57	2.32
34.72	1.65
1.32	0.87
9.32	1.76
23.6	1.23
23.1	1.5
16.22	0.87
17.05	0.67
17.29	1.22
10.93	1.98
12	0.46
22.06	0.87
8.79	2.1
43.2	1.2
32	0.61
13.1	0.98
26.58	0.65
16.47	2
30.04	2.26
23	0.48
13.4	1.58
11.22	7.8
8.37	1.53
15.5	0.54
21	0.26
12.62	0.43
12.4	1.23
14.7	7.4
12.4	0.98
2.41	0.54
9	1.43
12	0.34
23.85	1.87
18.53	1.34
31.15	2.14
21.4	1.2

31.18	1.2
14	0.87
21.34	1.3
48.29	1.49
13.39	0.76
14.87	1.9
12.33	2.34
21	0.75
23	1.32
11	2.7
12	2.1
6	0.85
12	0.99
12.18	0.61
13.81	2.31
21.83	0.14
19.01	1.54
12.93	1.32
42.47	1.23
11.42	2.99
22.06	1.54
70.56	0.87
13.4	1.43
27.19	0.95
20.95	2.1
16.77	1.75
10.71	0.16
15.02	0.18
15.82	1.54
17.48	0.23
269.9	5.5
20.95	1.54
16.27	3.2
16	0.61
19.02	0.87
12	1.67
15	0.99
13.78	2.68
21.17	0.87
12.4	0.87
11	0.54
32.22	2.32
43.89	0.79
14.42	0.79
23	2.42
19.01	4.6
25.57	0.79
27.7	0.98
16.35	1.65
8.23	6.3
21	0.45

10.7	1.43
6.36	0.66
12	0.98
23	0.87
29.59	5.3
2.17	0.98
22.84	2.97
87.39	2.32
21.73	0.32
13.49	1.76
9.76	0.46
32	1.43
6.36	7.4
12.1	0.66
12.3	1.98
47.14	1.32
23.4	0.54
10.15	1.45
25.32	76
43	0.54
15.61	0.09
13.83	5.43
19.57	0.34
14.84	0.76
23.64	3.3
15.07	2.1
12	1.54
8.79	0.87
6.36	0.39
16.3	1.98
21	0.43
21.17	1.54
43	0.26
10.69	0.98
6.77	0.45
19.96	0.98
28.22	2.1
4	1.54
25.63	0.7
21	1.54
12.4	0.46
21	1.23
12.3	1.32
12.3	1.4
23	0.98
4.53	0.54
9.01	5.3
14.95	1.43
9	0.43
222.3	0.67
14	0.26

12	4.35
18.33	5.3
15.65	0.46
22.83	0.65
22.08	0.05
370	1.4
9.3	0.09
19.23	1.65
14.79	2.11
15.28	13.34
2.53	0.46
9.16	1.43
34.81	1.76
14.41	0.9
23.1	2.53
107.6	0.86
9	0.46
11.44	2.56
12.4	0.66
13.93	1.65
6.36	1.967
9.35	0.39
10.69	5.5
51.64	1.43
40.87	1.43
6.36	1.75
15	1.54
34.81	2.24
43	1.4
9	2.1
32	0.99
12.8	0.87
39.33	7.4
23.1	0.54
121.6	1.98
16.77	1.65
25.23	1.32
20.27	0.4
52.55	0.61
17.25	2.43
11	0.46
5.6	1.54
8.4	1.12
23.06	5.3
13.32	1.43
14	1.4
60.13	1.43
8.24	0.54
16.87	6.3
21	0.87
21.43	1.56

23.85	1.2
14.54	1.2
370	0.61
49.88	2.54
12	1.98
25.63	1.3
12.82	0.67
5.6	1.76
12.37	0.41
18.33	1.43
18.34	1.54
22.53	0.61
32.1	1.06
23	2.7
9.21	0.76
2.52	0.67
10.68	1.98
11.2	1.54
20.95	2.01
20.38	0.93
16.26	1.32
14.41	2.4
14.87	1.54
5	1.54
18.8	1.53
12.3	1.86
54.32	2.01
12	1.36
21	1.43
14.14	2.1
39.16	1.68
13	1.98
14.91	1.98
8.9	1.65
15.32	2.99
15.65	0.34
60.13	1.43
43	0.97
14.74	0.87
10.47	0.98
21	1.54
17.25	0.26
21.32	1.89
34	4.6
32	7.8
14	1.65
35.89	0.58
14	1.2
23.59	1.3
31.56	0.26
67	2.32

184.7	1.54
9.01	1.43
15.65	1.45
17.29	0.7
38.44	1.43
8.34	1.43
1.32	1.43
17.07	0.26
18.16	3.21
16.76	0.87
215.4	1.54
21	2.12
14.96	0.54
21	0.26
20.95	0.98
13.4	0.54
6.55	1.87
21.71	2.32
16.04	0.53
15.43	1.43
12.3	2.65
12.3	1.87
6.15	2.43
17.96	0.87
23.6	5.3
8.14	0.46
21.3	1.54
87.39	0.46
19.91	2.54
43	1.98
21	0.87
13.2	0.76
9	1.75
20.94	2.32
11.67	5.3
14	0.98
11.5	0.78
149.2	1.36
9.98	0.12
4.53	1.54
32.12	0.26
102.2	0.87
17.82	8.55
102.2	1.23
13	0.97
134.9	0.43
27.1	0.98
5.5	0.23
13	1.2
22.6	0.66
11.87	0.87

23	0.34
21	1.06
25.63	2.32
107.6	1.98
27.94	1.43
17.05	0.98
18.4	0.34
128.3	2.32
10.88	0.76
38.56	0.98
14.46	1.22
11.44	1.54
25.35	0.87
41	1.43
12.4	1.23
23.4	0.23
32.38	3.21
24	4.34
3.21	0.97
7.5	1.32
15.52	0.87
19	2.12
14.87	5.3
11.44	0.61
15.5	1.23
13.83	1.53
27.15	1.3
32.12	0.79
13.4	1.54
10.69	1.3
13.26	0.7
13.43	0.75
12.4	0.96
38.44	5.5
17.37	6.3
12	0.98
32	2.32
23.72	0.98
9	2.54
22.65	2.7
13	1.3
11.78	1.98
12.4	0.34
15.36	1.32
32	0.54
18.33	5.5
13	1.22
12	1.54
12	0.87
22.6	0.66
10.7	0.46

13.2	1.98
11.64	1.34
22.53	1.93
13.83	1.67
20.2	1.54
12.32	1.23
8.21	1.14
16.74	0.5
23.15	0.97
15.93	0.66
32	0.43
43	1.34
3.21	1.43
21.3	1.87
23.61	1.34
9.8	1.23
23.4	0.98
32	0.61
11.16	0.76
19.32	0.87
12.1	0.87
11.83	1.32
15.24	2.12
12.15	2.43
21.21	2.13
12.32	3.56
21	0.3
18.71	1.54
19.5	0.23
16	1.2
11.67	1.06
21.3	1.47
38.61	1.65
43.12	1.43
23.2	5.6
29.59	1.68
101.6	0.87
12.27	2.32
21.17	0.76
19	76
39.96	0.54
67	1.75
21.17	0.98
14.41	0.54
43.77	0.54
102.2	2.31
19.29	1.43
29.12	1.29
35.61	1.23
60.13	0.56
43	0.98

11.44	1.43
23.77	1.67
14.73	1.67
15.4	1.16
28.7	1.16
15.18	0.66
3.6	1.76
10.69	1.5
10.2	1.3
9	0.57
11	1.3
14.33	0.98
18.34	0.46
11.36	0.786
23.63	1.42
13.62	1.53
17.25	0.54
10.67	1.32
12	2.32
12.53	1.54
100.5	0.97
12	0.76
11.67	1.17
23.4	1.75
14.24	0.68
22.76	1.3
11.64	0.99
4.53	0.66
10.63	1.54
23.41	1.4
13.19	1.2
13	0.97
48.29	1.65
13.39	1.3
10.93	0.87
18.24	0.99
1.32	1.43
34.81	0.43
14	0.64
19.4	2.12
13.77	1.32
21	1.54
18.53	0.26
30.04	0.97
15.32	0.87
15.95	0.97
26.89	5.5
12.98	0.76
12.95	0.21
21	2.32
19.01	2.43

101.6	1.3
43	1.54
14.95	0.87
208.5	0.87
12	1.3
41.73	1.4
7.95	0.46
3.21	0.9
19.29	0.34
1.21	0.54
10.69	0.54
13.92	7.4
43.21	1.3
15.88	0.34
17.87	1.09
13.39	0.43
8.5	1.54
13.55	0.78
15	0.43
18.94	2.97
34.21	1.34
21.45	5.6
22.96	2.12
40.63	2.31
13	0.97
22.53	0.87
21.3	0.61
43.2	0.54
61.86	0.76
64.39	0.9
21.21	0.87
16.47	3.56
22.06	0.65
125.1	1.54
121.5	0.87
15.5	1.3
22.6	1.45
29.27	1.56
52.55	0.26
32	0.67
14	0.87
5.32	0.64
20.95	2.7
14.95	1.43
101.6	0.87
8.79	1.68
13.43	0.65
14.87	0.87
13.2	0.26
18.93	1.43
12.43	0.48

12	1.76
10.68	5.6
32	2.87
10.7	1.2
13	0.56
25.63	0.39
11.67	0.72
17.25	0.9
22	1.34
17.21	1.74
11.36	0.26
94.47	1.43
22.77	0.98
16.88	1.76
14	1.54
24.32	3.56
13.26	1.23
21	2.32
4.53	1.98
17.08	0.34
17.69	2.54
16.76	1.54
5	0.97
15.81	0.59
22.33	1.54
12	1.54
25.63	0.99
43.77	2.99
12	0.34
20.95	1.65
17.83	1.9
22.44	2.43
7.63	1.87
21.17	1.43
102.2	1.3
8.14	3.2
8.97	2.54
20.41	3.4
17.91	1.54
11.67	0.87
10.7	4.6
12.3	0.93
12	1.54
21	1.76
25.28	2.32
24.56	2.78
15.52	0.87
87.39	1.54
23.6	0.76
28.11	1.14
199.2	0.43

12.4	1.3
21.83	5.6
32	1.2
43.77	0.46
12	1.23
13.06	1.3
22.5	1.32
87.39	0.86
14	0.56
32	2.99
16.56	1.3
32.12	0.76
4.41	0.87
15.5	5.3
54.74	0.54
52.55	1.4
19.57	0.94
32	0.72
12	1.54
12.72	1.43
11.22	0.98
13.59	1.76
39.43	1.3
12	0.43
15.5	0.86
12.32	0.97
23.32	1
15.52	0.99
23	1.43
48.29	0.61
17	0.54
39.79	0.41
16.47	1.06
9	1.42
28.71	0.66
27.19	9.19
13.39	1.87
12	1.76
15	0.66
23.13	1.35
15	5.43
15.6	0.86
27.94	1.32
8.23	2.7
2.94	1.16
21	3.2
